# Identification of novel genes involved in neutral lipid storage by quantitative trait loci analysis of *Saccharomyces cerevisiae*

**DOI:** 10.1186/s12864-021-07417-4

**Published:** 2021-02-09

**Authors:** Klavdija Pačnik, Mojca Ogrizović, Matthias Diepold, Tobias Eisenberg, Mia Žganjar, Gašper Žun, Beti Kužnik, Cene Gostinčar, Tomaž Curk, Uroš Petrovič, Klaus Natter

**Affiliations:** 1 Institute of Molecular Biosciences, NAWI Graz, University of Graz, Graz, Austria; 2 Department of Molecular and Biomedical Sciences, Jožef Stefan Institute, Ljubljana, Slovenia; 3 BioTechMed-Graz, Graz, Austria; 4 Field of Excellence BioHealth – University of Graz, Graz, Austria; 5 Faculty of Chemistry and Chemical Technology, University of Ljubljana, Ljubljana, Slovenia; 6 Department of Biology, Biotechnical Faculty, University of Ljubljana, Ljubljana, Slovenia; 7 Faculty of Computer and Information Science, University of Ljubljana, Ljubljana, Slovenia

**Keywords:** baker’s yeast, triacylglycerol, steryl esters, lipid metabolism, lipid droplet, polygenic trait, natural variation, QTL analysis

## Abstract

**Background:**

The accumulation of intracellular fat depots is a polygenic trait. Therefore, the extent of lipid storage in the individuals of a species covers a broad range and is determined by many genetic factors. Quantitative trait loci analysis can be used to identify those genetic differences between two strains of the same species that are responsible for the differences in a given phenotype. We used this method and complementary approaches to identify genes in the yeast *Saccharomyces cerevisiae* that are involved in neutral lipid storage.

**Results:**

We selected two yeast strains, the laboratory strain BY4741 and the wine yeast AWRI1631, with a more than two-fold difference in neutral lipid content. After crossing, sporulation and germination, we used fluorescence activated cell sorting to isolate a subpopulation of cells with the highest neutral lipid content from the pool of segregants. Whole genome sequencing of this subpopulation and of the unsorted pool of segregants implicated several loci that are involved in lipid accumulation. Three of the identified genes, *PIG1*, *PHO23* and *RML2*, were investigated in more detail. Deletions of these genes and the exchange of the alleles between the two parental strains confirmed that the encoded proteins contribute to neutral lipid storage in *S. cerevisiae* and that *PIG1*, *PHO23* and *RML2* are the major causative genes. Backcrossing of one of the segregants with the parental strains for seven generations revealed additional regions in the genomes of both strains with potential causative genes for the high lipid accumulation phenotype.

**Conclusions:**

We identified several genes that contribute to the phenotype of lipid accumulation in an allele-specific manner. Surprisingly, no allelic variations of genes with known functions in lipid metabolism were found, indicating that the level of storage lipid accumulation is determined by many cellular processes that are not directly related to lipid metabolism.

**Supplementary Information:**

The online version contains supplementary material available at 10.1186/s12864-021-07417-4.

## Background

The pathways for the biosynthesis of triacylglycerol (TAG) and steryl esters (SE) are highly conserved from unicellular yeasts to humans. The main roles of these lipids are the storage of energy and of precursors for the synthesis and for the remodeling of membrane lipids. In addition, the sequestration of lipids into intracellular lipid droplets (LD) is regarded as a means to avoid the accumulation of membranes or toxic effects of lipids when they are synthesized or taken up in excess of the cellular demand [1, 2]. The storage of neutral lipids (NL) is regulated on many levels and a large number of genes have an influence on lipid accumulation. Therefore, the accumulation of storage lipids is a quantitative trait and, although some of the regulatory mechanisms that control the NL levels are characterized, it has to be assumed that only a small number of the involved genes are known [3, 4].

Like all eukaryotes, the model yeast *Saccharomyces cerevisiae* is able to synthesize and store NL. As expected for a polygenic trait, the variability of this phenotype among different strains is high [5]. The enzymes of all lipid pathways are mostly characterized [6] and many examples of changes in lipid storage in response to altered expression, deletion or interference with posttranslational control of these genes/proteins have been reported. However, the results from genome-wide deletion studies suggest that a large number of other genes, many of them without reported functions in lipid metabolism, influence the NL content of yeast [4, 7, 8]. In addition to loss-of-function mutations, nucleotide polymorphisms can contribute to differences in the NL content if they result in different expression levels or changes in activity or stability of a protein, but only little is known about the quantitative contribution of such allelic variations to NL metabolism and storage.

The development of quantitative trait loci (QTL) analysis has paved the way to identify the causal alleles contributing to non-Mendelian traits. This method allows for the identification of genetic loci that are responsible for the phenotypical differences between two individuals and has been successfully applied in many organisms. Since the first QTL studies in *S. cerevisiae* [9, 10], this method has been further optimized. The availability of affordable whole genome sequencing (WGS) techniques allows now for the sequencing of large pools of segregants, a method called X-QTL [11]. The power of this technique in the analysis of polygenic traits has been demonstrated in many studies. Prominent examples are the mapping of causal alleles for tolerance to chemicals [11, 12] or to high [13, 14] or low [15] temperature, the identification of longevity alleles [16, 17], the analysis of the genetic basis for variability of growth [18], and the mapping of QTLs contributing to the production of volatile compounds [19] and to the control of sporulation [20].

In this study, we investigated two *S. cerevisiae* strains that have different NL storage capacities, the widely used laboratory strain BY4741 [21] and AWRI1631 [22], a strain used in the wine industry. The genome of AWRI1631 has been sequenced and more than 68,000 individual nucleotide variations have been found in comparison with the reference genome of S288c [22], from which BY4741 is derived. We performed a QTL study with the two strains to identify the causal alleles for high NL content. Most of the loci that were enriched in the segregants with high NL content had no known connection to the biochemical pathways contributing to lipid synthesis, storage or degradation. Three of the identified genes were analyzed in more detail, to confirm the validity of the approach and to quantitate the contribution of the causative alleles to lipid accumulation in yeast.

## Results

### An X-QTL study identifies several loci potentially contributing to TAG accumulation

**Fig. 1:**
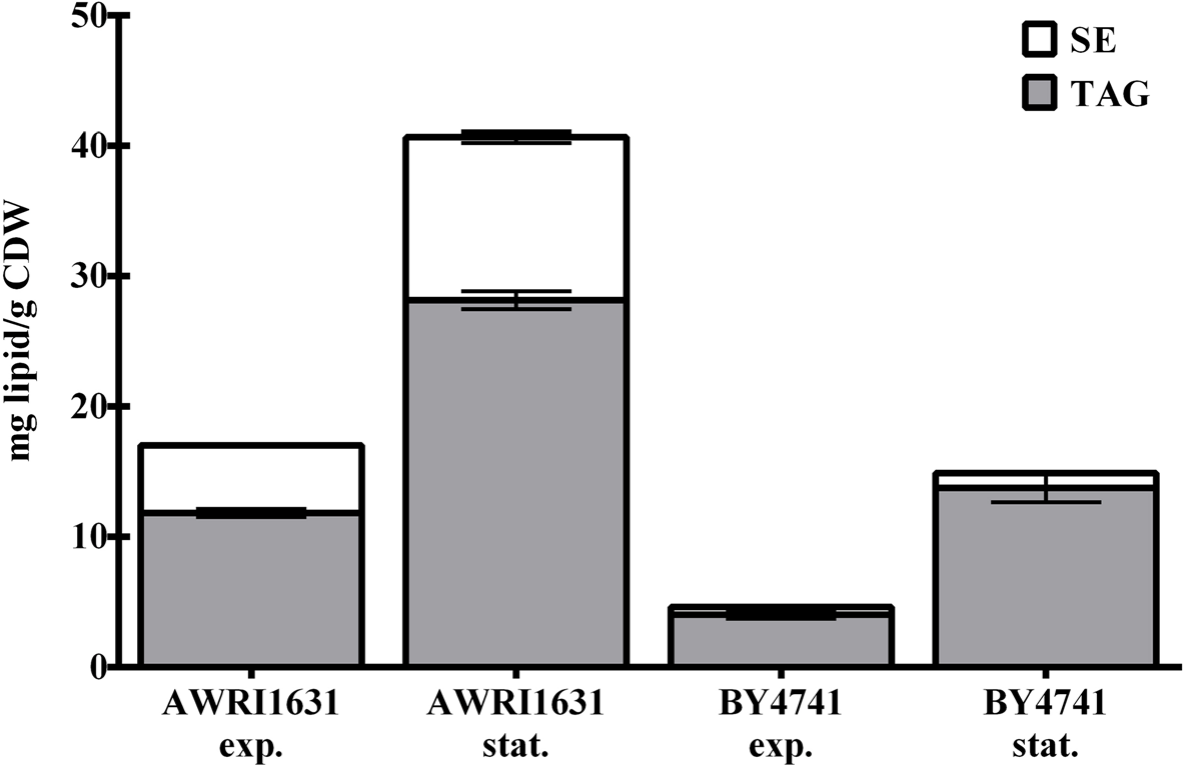
TAG and SE content of the two yeast strains AWRI1631 and BY4741. The cultures were harvested during exponential growth (exp.) or in stationary phase after 48 h (stat.) of cultivation in minimal medium. The data are the means from a minimum of six independent experiments and their standard deviations. The *p*-values, calculated with a two-tailed t-test, were < 0.001 for the comparisons of the two strains in both exponential and stationary phase and for both compounds, TAG and SE.

**Fig. 2:**
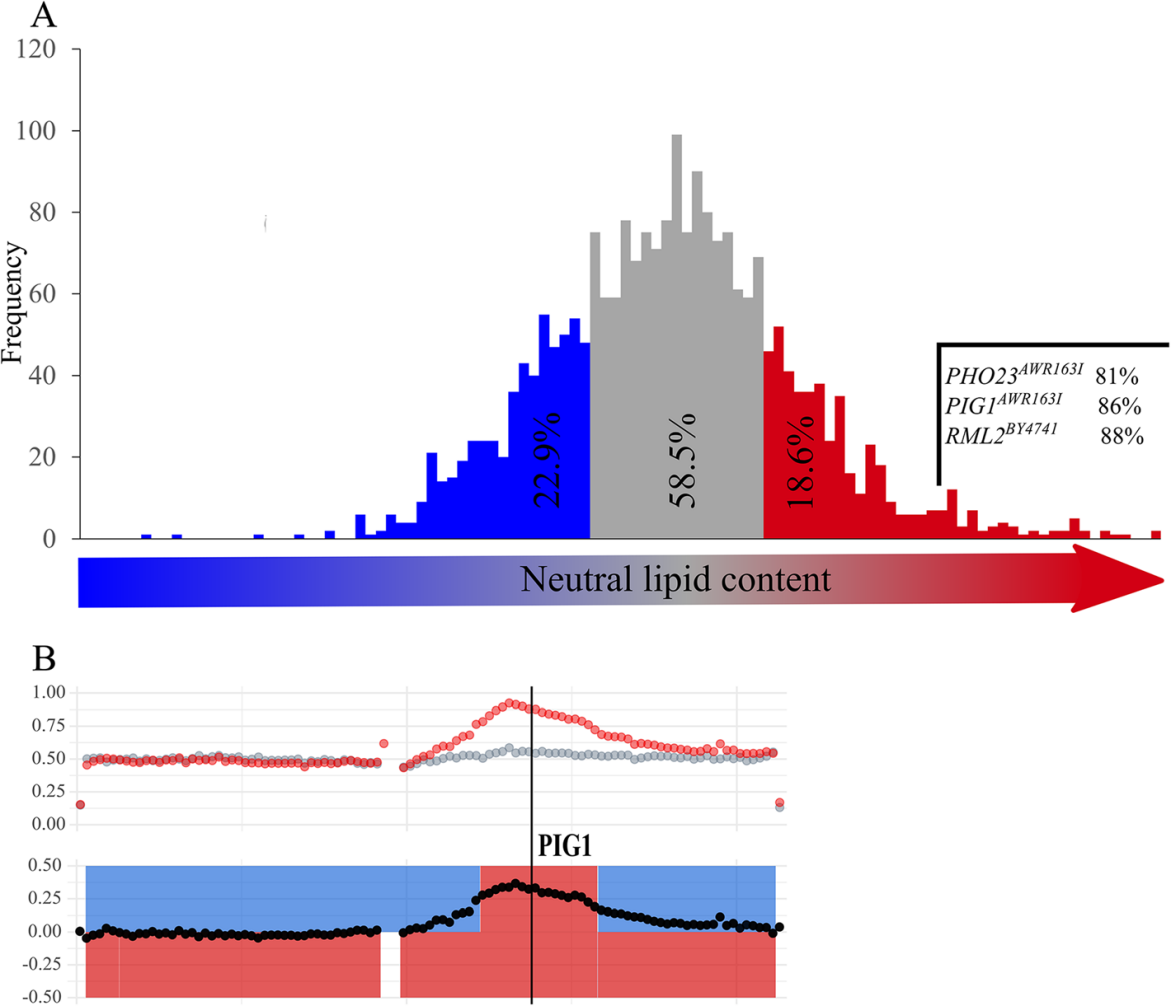
Analysis of segregants from crossing BY4741 with AWRI1631. **a**: Frequency distribution of fluorescence intensity of 2288 F1 segregants. The segregants with lower fluorescence than the BY4741 parental strain are shown in blue, the segregants with higher values than the AWRI1631 parental strain in red, and the segregants with intermediate intensity are depicted in gray. The frequencies of alleles that are beneficial for high NL content are shown for a subset of 43 out of the 60 segregants with the highest FI. **b**: WGS data of a section of chromosome XII, including the *PIG1* peak (see Fig. S1 for the analysis of all chromosomes). The figure shows the median ratios between the frequencies of BY4741 and AWRI1631 parental strain-derived SNVs in the X-QTL analysis. Red points: selected subpopulation with high [NL]. Gray points: non-selected population with average [NL]. Each point shows a median AWRI1631:BY4741 ratio for all SNVs in a window of 10,000 bp. Black points: difference between the populations: higher abundance of the red than of the gray signal indicates that this region is enriched for AWRI1631 sequences in the population with high NL content. The gap in the signal marks a region derived exclusively from the BY4741 parent, i.e. a region with no SNVs calls relative to the BY4741 variant calling reference. Shading denotes the parental origin of the genomic region in the F7 generation of the backcrossing experiment selecting for high NL content (BY4741 – blue; AWRI1631 – red) in the BY lineage (upper ribbon) and in the AWRI lineage (lower ribbon)

The major storage lipids in yeast are TAG and SE. We determined the content of TAG and SE for both exponentially growing and stationary cultures of two strains, the laboratory strain BY4741 and AWRI1631, an industrial wine strain, in which the *HO* locus was deleted for stable haploid propagation [22]. In stationary phase, i.e. under starvation conditions, AWRI1631 and BY4741 accumulated 28.1 ± 0.7 mg and 13.8 ± 1.1 mg TAG per g cell dry weight (CDW), respectively. The levels of SE were lower than the TAG levels but the difference between the two strains was even more pronounced, with 12.5 ± 0.4 mg/g in AWRI1631 and 1.10 ± 0.10 mg/g in BY4741. In total, AWRI1631 accumulated ca. 41 mg of NL, whereas only ca. 15 mg were measured in BY4741 (Fig. 1). Because we obtained the same ratio between the two strains in the exponential growth phase, we restricted our further analysis to cultures in the stationary phase, where storage lipids are more abundant than during growth. To identify the genes that are responsible for the increased lipid accumulation in AWRI1631, we first performed an X-QTL study. After crossing the two parental strains, sporulation of the hybrid and germination of their haploid progeny, we picked 2288 single colonies into 96 well microtiter plates to analyze the distribution of the NL content in the F1 generation. For this experiment, we used an assay based on Nile Red fluorescence intensity (FI), which correlates with NL content, as described in ‘Methods’. As shown in Fig. 2a, the FI in these cells covers a broad range with an almost normal distribution, indicating that many genes contribute to the stimulation or repression of lipid storage. More than 40% of the progeny showed a heterosis effect: 22.9% of the strains had a lower FI than the BY4741 parental strain, and 18.6% of the segregants showed stronger fluorescence than the AWRI1631 parental strain. Next, approximately 2.5 × 10^8^ colonies of haploid segregants were scraped off the plates and pooled. An aliquot of this average population, corresponding to at least 1.5 × 10^7^ genetically distinct segregants, was sorted by fluorescence activated cell sorting (FACS), to separate a subpopulation with strong Nile Red fluorescence, indicating high NL content (Supplemental Fig. S1). Both, the average population and the subpopulation with high NL content were subjected to tiling DNA microarray-based analysis for genome-wide detection of single nucleotide variations (SNVs) with SNPscanner. This algorithm calculates a prediction signal for the presence of a SNV at a nucleotide position using measurements from a single hybridization to a whole-genome DNA microarray [23]. A higher prediction signal thus indicates a higher frequency of alleles derived from the AWRI1631 strain, as the probes on the microarray matched the BY4741 strain genomic sequence. Furthermore, whole genome sequencing of the pools of segregants in these two subpopulations was performed to obtain a better resolution of the SNVs in the subpopulation of segregants with high NL content compared to the average population. The mean of the coverage for the average pool was 738-fold, and for the high NL content pool it was 1407-fold. As an example, WGS results for the two populations are shown for chromosome XII in Fig. 2b. The data for all chromosomes are shown in Supplemental Fig. S2.

Table 1 lists the eight genes whose genome locations were identified using the DNA microarray-based method, and for which WGS analysis confirmed non-silent SNVs with the highest bias for one of the parental alleles in the corresponding loci of the subpopulation with high NL content. For seven of these genes (*PIG1*, *PHO23*, *AQR1*, *PML39*, *SWH1*, *AFI1* and *ZDS2*) WGS analysis confirmed that the AWRI1631 strain allele was enriched in the subpopulation with high NL content. In the case of *RML2*, however, the enrichment of the BY4741 allele in this population could be the consequence of linkage with the *CAN1* locus, which was one of the selection markers, and we therefore could not conclude which allele is beneficial in terms of NL content.

**Table 1:** List of potential quantitative trait loci, based on the genome-wide detection of polymorphisms at nucleotide resolution with DNA microarrays and WGS

Chromosome	Gene/s	SNPscanner prediction signal	% of AWRI1631-derived SNVs in the high NL content subpopulation	# of non-silent SNVs in ORF	# of SNVs in the 5′-upstream region (500 bp)
XII	*PIG1*	3.91	86–95	14	0
XIV	*PHO23*	2.21	87–91	1	7
XIV	*AQR1*	1.57	82–90	3	4
XIII	*PML39*	1.18	77–81	2	3
I	*SWH1/YAT1*^*a*^	0.97	46–73	6	3
V	*RML2*	0.95	18–28	1	3
XV	*AFI1*	0.84	66–75	5	1
XIII	*ZDS2*	0.81	73–79	7	3

^a^Resolution too low for identification of one single gene

### Changes in TAG and SE storage upon deletion of selected potentially causative genes

**Fig. 3:**
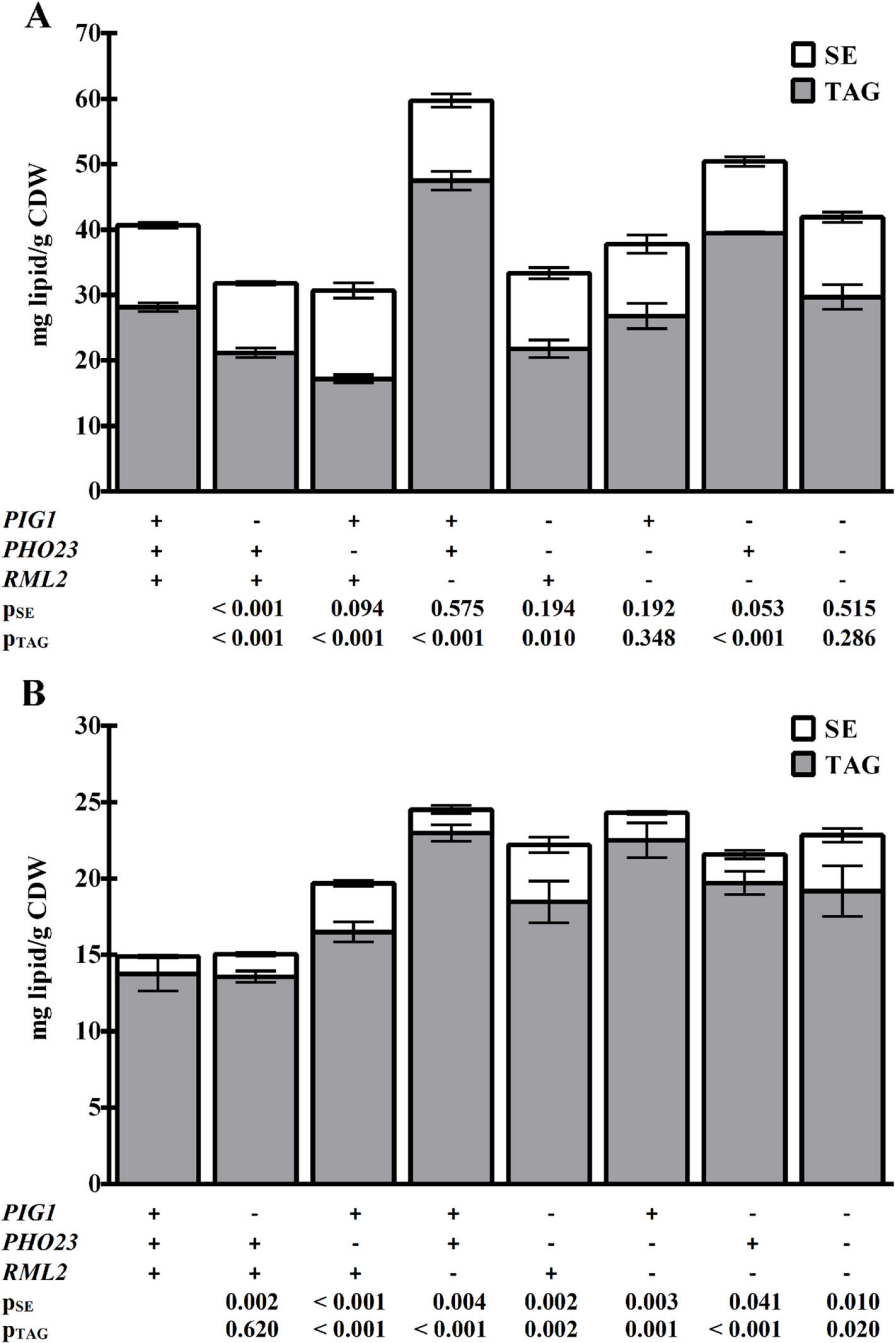
Neutral lipid analysis of deletion mutants. TAG and SE content of the AWRI1631 (panel **a**) and the BY4741 (panel **b**) strains, deleted for *PIG1*, *PHO23* or *RML2*, and combinations thereof. These results confirm that the proteins encoded by these three genes are involved in NL metabolism, with varying influence in the two strain backgrounds. The strains were cultivated in minimal medium for 48 h. The data are the means from a minimum of three independent experiments and their standard deviations. The *p-*values are the results of a two-tailed t-test comparing the respective mutant with the wild-type.

Based on the results of the X-QTL study, three genes were selected for further analysis and quantification of their contribution to NL storage. *PIG1* and *PHO23* were selected as the most likely causative genes, according to the SNPscanner prediction signals and the frequency of AWRI1631-derived variants in the subpopulation of segregants with high NL content. *RML2* was included because the SNPscanner analysis proposed the existence of a minor QTL in the vicinity of the *CAN1* locus. This locus must be inherited from the BY4741 parental strain (*can1∆*) in the selected segregants, due to their cultivation on canavanine-containing media. The most likely causative gene in this region was *RML2,* with a distance of 26.2 kbp (corresponding to 9 cM according to [24]) to the *CAN1* locus. In addition, a mutant allele of Rml2p was shown to be deficient in oleic acid utilization [25], suggesting a connection to lipid metabolism. However, neither *RML2* nor any of the other QTLs listed in Table 1 have been implicated in NL storage so far. Using SNV-specific PCR, we genotyped a subset of 43 out of the 60 strains with the highest NL content among the 2288 single segregants, according to the Nile Red-based assay. This analysis showed that 88% carried the *RML2* allele from the BY4741 parental strain, whereas 86 and 81% had the AWRI1631 *PIG1* and *PHO23* alleles, respectively (Fig. 2a), suggesting that these three genes indeed play a role in lipid storage metabolism and that the *RML2* allele of the BY4741 strain is beneficial for NL accumulation. To test their quantitative contribution to NL accumulation and to investigate how QTL results are reflected in the NL content of mutant strains, single deletion mutants for the three genes in both strain backgrounds were constructed and subjected to lipid extraction and quantitative analysis of TAG and SE after growth into stationary phase. For the *pig1*∆ strains, we found that the TAG and SE contents in AWRI1631 were reduced by 25 and 15%, respectively, whereas NL storage in BY4741 was only marginally affected by the loss of Pig1p. The loss of Pho23p function resulted in a drop by 39%, as compared to the wild-type TAG level in the AWRI1631 strain, whereas the SE content remained unchanged. In contrast, the deletion of *PHO23* in BY4741 resulted in a slight increase in TAG content but in almost three times more SE than in the wild-type, with an overall increase of NL by 32%. The deletion of *RML2* resulted in a strong increase in TAG content by 69 and 67% in AWRI1631 and BY4741, respectively. The SE content remained at the wild-type level in the AWRI1631 *rml2∆* strain, whereas it increased by 34% in the BY4741 background (Fig. 3).

Pho23p was characterized as a component of the Rpd3L histone deacetylase complex [26]. To answer the question whether the opposite effect of the deletion of *PHO23* in the two strain backgrounds is specific for this gene or a result of a reduction or loss of function of Rpd3L, we analyzed several mutants that were deleted for other components of the Rpd3L complex. To avoid a possible bias due to growth defects, we selected four mutants - *rpd3∆, sap30∆, sds3∆* and *rxt2∆* - for which no such phenotypes were reported. Indeed, we confirmed wild-type-like growth for these mutants and the same trend as in the *pho23*∆ background with regard to lipid storage, i.e. higher NL content in the BY4741 background and reduced levels in AWRI1631. The only exception from this rule was the strain deleted for *RPD3* in BY4741, which had a slightly lower NL content than the wild-type (Supplemental Fig. S3). These data suggest that the Rpd3L complex plays different roles in these two strains with respect to NL storage. It should be noted that two out of the four tested genes, *SDS3* and *RXT2*, bear variations that result in differences between the two parental strains on the protein level. Hence, the different effect of the complex on lipid storage might be the result of several proteins with slightly altered functionality.

In the case of *RML2*, which was characterized as a component of the mitochondrial large ribosomal subunit, we randomly selected four other proteins of this complex, Mrpl3p, Mrp7p, Mrpl8p and Mrpl49. Lipid analysis of the respective knock-out strains showed that none of these mutants accumulated TAG in similar amounts as the strain deleted for *RML2* (Supplemental Fig. S4). In all four mutants the NL content was slightly higher than in the wild-type, but we assume that this change is a consequence of the growth defect of these strains, due to the loss of functional mitochondria. Based on these results, we assume that the role of Rml2p in lipid metabolism might be independent of its function as a ribosomal protein.

Finally, we deleted *GAC1*, the second gene besides Pig1p encoding a protein tethering the protein phosphatase Glc7p to the glycogen synthase Gsy2p, in the AWRI1631 strain. However, the NL content in this mutant was not significantly different from the wild-type value (Supplemental Fig. S5). Therefore, the reduced NL content of AWRI1631 *pig1∆* is not a general consequence of altered tethering of Glc7p to Gsy2p, but rather the result of another function of Pig1p in AWRI1631.

Furthermore, we constructed double and triple deletion mutants of the three genes, to investigate possible genetic interactions. None of these strains showed a growth defect, except for the slightly slower growth of the mutants with a deletion of *RML2*. Lipid analyses indicated a genetic interaction effect between *PHO23* and *PIG1* in AWRI1631 because the TAG content of the double mutant was between that of the wild-type strain and the two single mutants, whereas an additive effect on the TAG content, and therefore a significantly lower content than in the two single mutants, would have been expected in the case of two independent genes (Fig. 3).

### Effects of allele substitutions

**Fig. 4:**
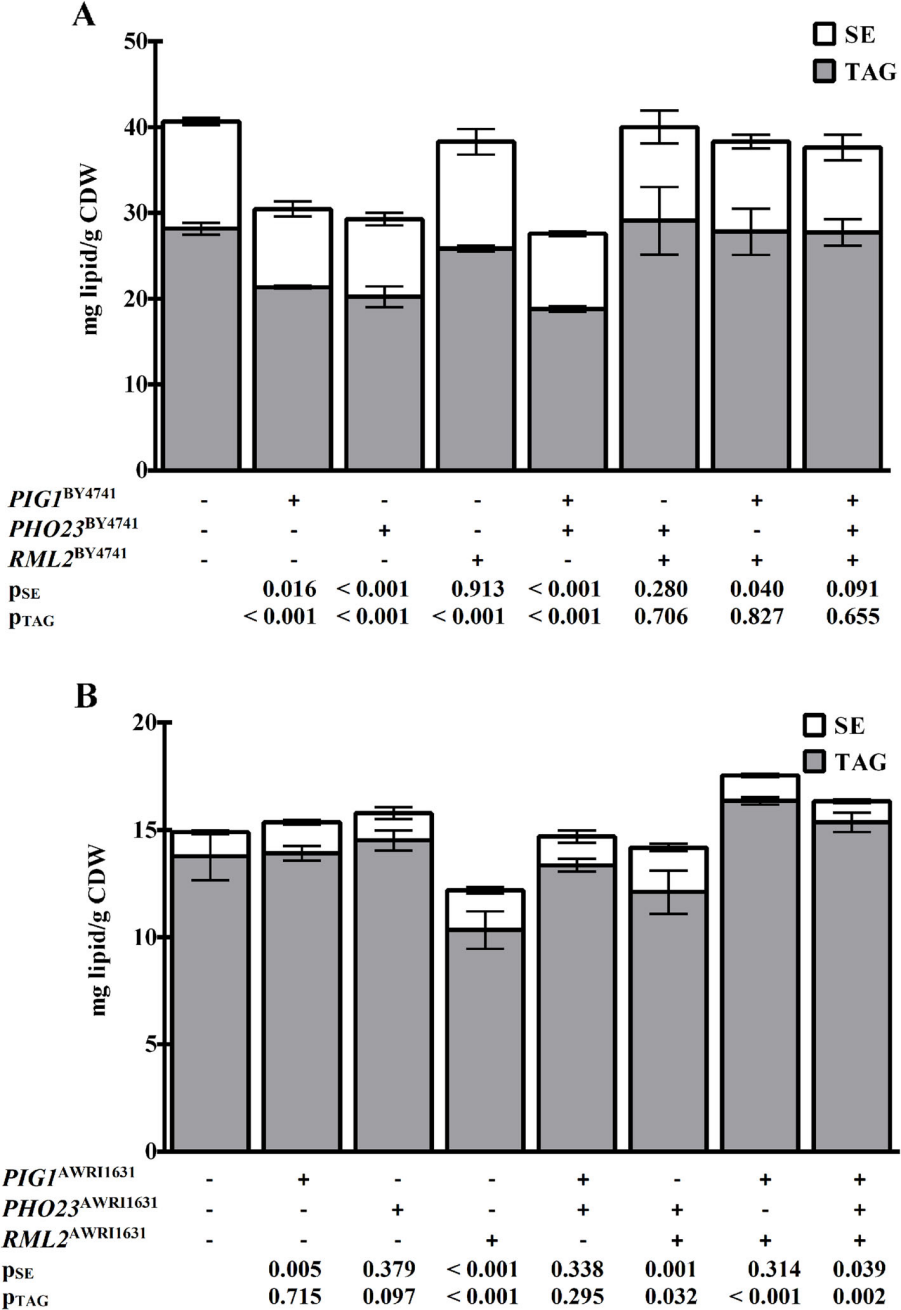
Neutral lipid analysis of substitution mutants. TAG and SE content of the AWRI1631 (panel **a**) and the BY4741 (panel **b**) mutant strains. The genes *PIG1, PHO23* or *RML2* and combinations of these genes are replaced with the alleles from the other parent strain. The mutants were cultivated in minimal medium for 48 h. The data are the means from a minimum of three independent experiments and their standard deviations. The *p-*values are the results of a two-tailed t-test comparing the respective mutant with the wild-type.

The NL content analyses in the deletion strains support our findings from the QTL study that all three proteins are connected to NL storage to varying degrees and with different effects. To confirm the importance of the allelic variations between the two strains, we substituted the three protein coding regions of the genes in both genetic backgrounds with the alleles from the other parental strain. This allele swapping resulted in reduced NL content in AWRI1631 with substitutions of *PIG1* (− 25%) or *PHO23* (− 28%), as expected from the enrichment of the respective AWRI1631 alleles in the subpopulation with high NL content in the QTL study. However, no effect was observed for the substitution of *RML2* (Fig. 4a). On the other hand, the substitution of *PIG1* or *PHO23* in the BY4741 background did not affect NL accumulation, whereas the *RML2* allele of AWRI1631 caused a drop in NL content by 18% (Fig. 4b).

These results indicated that the three genes are indeed involved in NL storage, but that their function depends on one or more other factors that are present only in one parental strain. To support this assumption, we selected 8 haploid segregants with high NL content from the 2288 strains derived from the crossing AWRI1631xBY4741, which bore the *PIG1* allele from AWRI1631. The *PIG1*^*AWRI1631*^ allele was replaced in these segregants with the allele from BY4741. The resulting strains had on average 6% lower NL content, with statistically significant difference (two-tailed t-test: *p* = 6.6 × 10^− 4^, Supplemental Fig. S6). Importantly, the effect of the substitution showed a rather high variation, indicating that the quantitative contribution of Pig1p^AWRI1631^ to NL content depends on other factors that were present in only part of the segregants.

To assess the potential combined effects of the alleles, we first constructed the double substituted strain AWRI1631 with *PIG1*^*BY4741*^ *PHO23*^*BY4741*^ alleles, resulting in a strain with less efficient alleles for NL accumulation in the genetic background of the parental strain with higher NL content. Indeed, the TAG content of this mutant dropped to 19 mg/g_CDW_, which is only 36% higher than the TAG content of BY4741 (Fig. 4a). Surprisingly, the additional substitution of *RML2* in this strain resulted in a strong increase of the TAG content, although *RML2* substitution alone did not show any effect in AWRI1631. Thus, presence of the *RML2*^*BY4741*^ allele in the AWRI1631 genetic background masked the phenotypic effect of the substitution of the *PIG1* and *PHO23* alleles, indicating a genetic interaction. Also, comparison of the single- and double-swapped strains in the AWRI1631 background showed that the contribution of the *PIG1* and *PHO23* alleles to NL content is non-additive. On the other hand, the TAG level of the BY4741 *PIG1*^*AWRI1631*^ *PHO23*^*AWRI1631*^ strain was not different from the wild-type BY4741 strain, despite the fact that these two alleles are apparently beneficial for high NL content. This result suggests that (an)other allele(s) in the AWRI1631 genetic background is (are) required for them to exert an increase in NL content. In the BY4741 background the strongest genetic interaction was observed between *PIG1*^*AWR1631*^ and *RML2*^*AWRI1631*^ alleles, since their double substitution caused an increase in NL content, whereas the single *RML2* and *PIG1* substitutions decreased or had no effect on the NL content, respectively.

### Contributions of other causative genetic elements: backcrossing

**Fig. 5:**
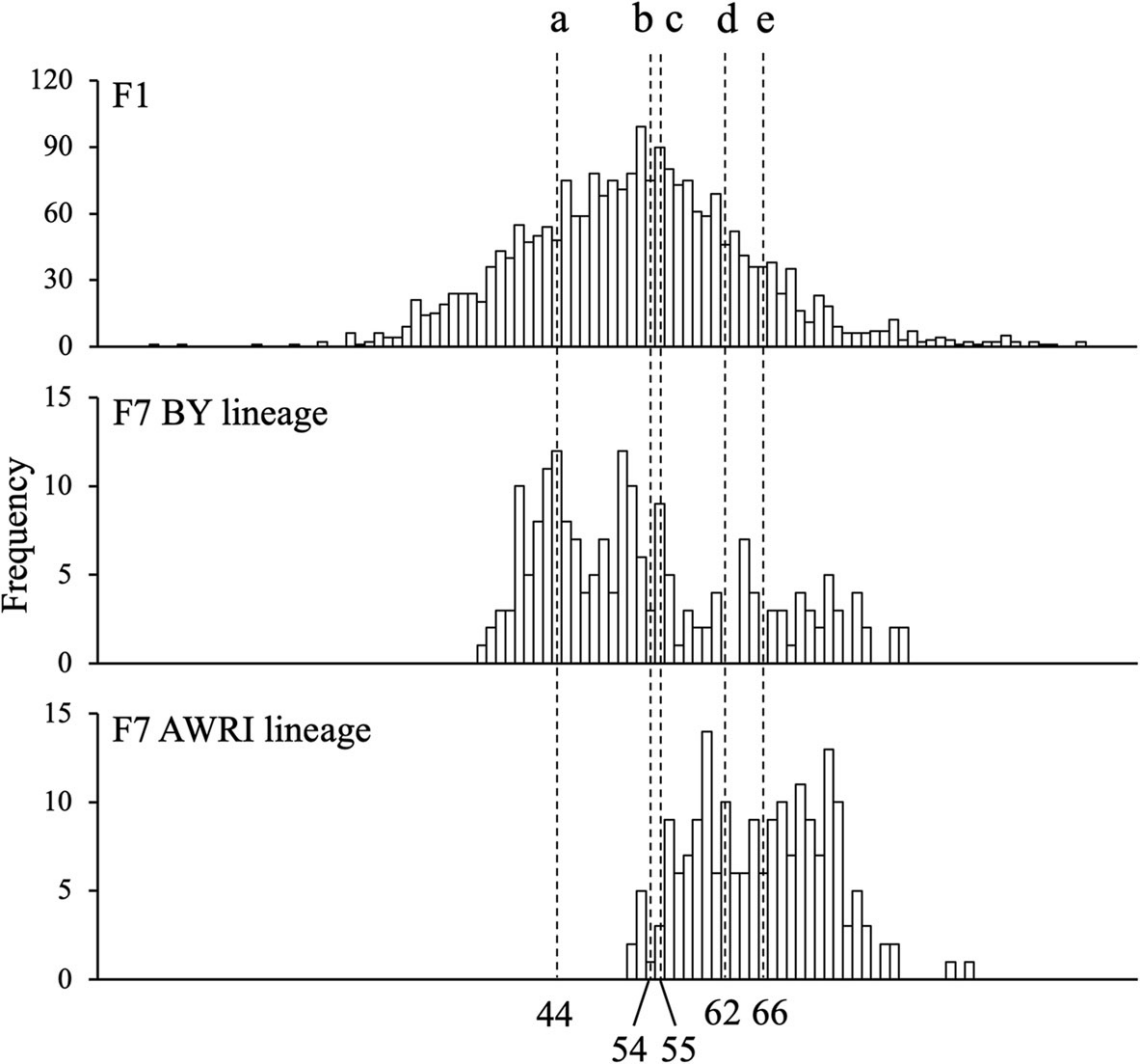
Neutral lipid content of the BY and AWRI lineages after backcrossing. The figure shows the frequency distribution of the fluorescence intensity of Nile Red, which correlates with the NL content, of the F1 generation (upper panel), the F7 generation of the BY lineage (middle panel) and the F7 generation of the AWRI lineage (lower panel). The FI of the segregants was normalized to the value of the parental strain BY4741 and according to the log_2_ value of the normalized intensity the segregants were distributed into 100 intervals. Dotted lines denote some important classes: line a (44^th^ class; log_2_(ratio of [FI]) = 0) – segregants with the same FI as in the BY4741 parental strain; line b (54^th^ class; log_2_(ratio of [FI]) = 0.37) – the average class of the F1 generation; line c (55^th^ class; log_2_(ratio of [FI]) = 0.40) – the average class of the F7 generation of the BY lineage; line d (62^nd^ class; log_2_(ratio of [FI]) = 0.66) – segregants with the same FI content as in the AWRI1631 parental strain; line e (66^th^ class; log_2_(ratio of [FI]) = 0.81) – the average class of the F7 generation of the AWRI lineage

While *PIG1*, *PHO23* and *RML2* were confirmed as causative genes for NL content in yeast, they did not explain all the variation between the parental strains. To identify additional potentially causative genetic elements for yeast NL content, we performed backcrossing experiments for 7 generations with both parental strains. The strain with the highest NL content (107 mg/g_CDW_ according to TLC analysis, corresponding to 263% of the AWRI1631 parental strain) from the F1 generation, which also had reproducibly high levels of NL according to the Nile Red method, was crossed with either of the parental strains to obtain the F2 generation for each lineage. In every generation, the strain with the highest fluorescence intensity of each lineage was selected and backcrossed with the same parental strain as in the previous step. The distributions of the 192 segregants of the F7 generation from both lineages, compared to the 2288 segregants of the F1 generation, are shown in Fig. 5. The average FI of the F7 generation of the BY lineage was similar to the average FI of the segregants in the F1 generation, whereas in the AWRI lineage the average FI of the F7 generation was 36% higher than in the F1 generation. The average FI of the back-crossed populations was higher than in the corresponding parental strains in both lineages (Fig. 5), but this difference was more pronounced in the F7 generation of the BY lineage than in the AWRI lineage. Therefore, the potential to increase the NL content is higher in the BY4741 parent than in the one that has already a high NL content and the remaining genetic background from the AWRI1631 parent in the BY line after seven generations of backcrossing has a higher impact on NL content than the remaining BY4741 genome in the AWRI line.

To identify these remaining regions of the other parent strain, the segregants with the highest NL content from every generation of both lineages, which were also used for the next backcrossing to the lineage’s parental strain, had their genomes sequenced. Supplemental Fig. S7 shows the genomes of the corresponding strains from F2 to F7. This selection and backcrossing procedure resulted in strains with between 3.2 and 6.5% of the genome from the parental strain of the other lineage (Supplemental Fig. S7). These values are significantly higher than the value of 0.8% that would have been expected in the 7th generation without any selection, indicating at least partial causality of the retained regions for the selected trait. In the case of the BY lineage, regions from the AWRI1631 parental strain included the whole QTL on chromosome XII, containing *PIG1* (Fig. 2b), and most of the sequence of chromosome I, including the QTL for which the resolution was not sufficient to distinguish between *SWH1* and *YAT1* as the causative gene (Table 1). To determine the causative gene in this QTL, we deleted *SWH1* and *YAT1* in both strain backgrounds. Whereas no changes in NL content were found for the *yat1*∆ strains, we obtained a moderate increase of 11% upon deletion of *SWH1* in BY4741 and a decrease of 23% in the AWRI1631 parent strain. Swh1p is a member of the family of oxysterol-binding proteins. Besides a domain for sterol-binding, it contains a motif for binding of phosphatidylinositol lipids and an ankyrin repeat region that is responsible for targeting of the protein to the nucleus-vacuole junction. Although its exact function is still not known, there is strong evidence that Swh1p plays a role in sterol and membrane lipid homeostasis [27, 28]. The results from the present study suggest that Swh1p is also involved in the regulation of the NL content of *S. cerevisiae*, which might explain the detection of this locus in the QTL study as well as its retention in the backcrossing experiment (Supplemental Fig. S8). On the other hand, the QTL on chromosome XIV including the *PHO23*^*AWRI1631*^ allele was lost in F5. In addition, several regions, on chromosomes I, II, III, VIII, X, XI and XV, were retained from the parental strain with higher NL content. In the AWRI lineage, apart from the marker regions around *LYP1* (chromosome XIV) and *CAN1* (chromosome V), which were expected to be retained due to the selection procedure for *MATa* haploids in each round of backcrossing, six regions, on chromosomes II, IV, VI, VIII, IX and XV from the BY4741 parental strain were retained. All regions and the genes in these regions with non-silent SNVs with respect to the other parental strain are listed in Supplemental Tables S2 and S3.

It should be noted that we found during the analyses of whole-genome sequences a VIII-t-XVI translocation in the genome of the AWRI1631 parental strain, which has not been previously described in this strain, but is known to be present in a number of wine strains [29]. This translocation was found also in the F1 segregant with the highest NL content, i.e. the founder of both backcrossing lineages, which however had also the complete chromosome XVI and thus effective duplication of the translocated region (Supplemental Fig. S9). In the process of backcrossing this duplication was retained in the BY lineage, whereas the karyotype was reverted to that of the AWRI1631 strain in the selected strain in the F5 generation (data not shown). For better comparability, the chromosomes for both lineages are presented in the organization according to the S288c reference genome.

## Discussion

In this study, we aimed at the identification of genes that contribute to the differences in neutral lipid storage in the two yeast strains BY4741 and AWRI1631. The sorting of cells as a function of fluorescence intensity of a lipophilic dye allowed for the enrichment of a large number of segregants with a phenotype that is not selectable by growth conditions, the commonly used approach for the selection of segregants with a desired phenotype in QTL studies. WGS and comparison of the total pool of segregants and of the FACS-sorted subpopulation revealed several alleles that were enriched in the latter, and follow-up experiments confirmed the involvement of three genes, *PIG1*, *PHO23* and *RML2*, in the regulation of NL storage. We determined the NL content on different levels, first, in a high-throughput assay using FACS, second, in a medium-throughput microtiter plate-based fluorescence assay and third, in cultures grown in shake flasks under optimal growth conditions by quantitative lipid analysis. Despite the lower accuracy of the high-throughput methods, due to higher variations in fluorescence intensity and bias derived from non-optimal cultivation conditions, the enrichment of the *PIG1* and *PHO23* alleles from AWRI1631 and of *RML2* from BY4741 in both assays and the confirmation of these genes with biochemical analysis of the deletion and substitution mutants demonstrates the potential of the presented approach to identify QTLs involved in NL accumulation.

For genotyping, two methods were used, hybridization on tiling DNA microarrays and WGS of pools of segregants. The combination of the results of the two methods enabled us to identify *PIG1* and *PHO23* as causative genes for NL storage, despite broad peaks in these two regions that included several genes. For example, the QTL on chromosome XIV had a peak overlapping with the gene *MKT1*, approximately 27 kbp away from the *PHO23* locus. However, SNPscanner scores peaked at the *PHO23* locus, indicating the causality of this gene. Since *MKT1* has been found to be causal for a variety of different polygenic traits (e.g. [30–32]), we deleted *MKT1* in both strain backgrounds and found no changes in the NL content of the mutants (data not shown), thus confirming that in the case of NL storage *MKT1* is not causal.

The crossing of AWRI1631 with BY4741 resulted in a strong heterosis effect, with segregants storing either more NL than the ‘fat’ parental strain, AWRI1631, or less than the ‘slim’ BY4741. This means that some of the genes of BY4741 are more efficient with regard to high NL accumulation than the alleles from the AWRI1631 strain background. Such a positive effect of alleles from the parent strain with the weak phenotype has also been reported in other studies [9, 33, 34]. In this work, *RML2* was identified as such a BY4741-specific allele with a beneficial effect on NL accumulation. The deletion of *RML2* resulted in an approximately 70% increase of the cellular TAG content, suggesting that Rml2p contributes to a process that negatively regulates TAG accumulation, with the protein variant from AWRI1631 being more efficient in this role than Rml2p from BY4741.

All loci that were identified during the QTL procedure (Table 1) have been reported to have functions that are not related to lipid metabolism. This result suggests that many perturbations throughout the metabolic and regulatory network of the cell, either through gene deletions or allelic variation, can have an impact on storage lipid metabolism. It is noteworthy that many of the genes encoding proteins of lipid metabolic pathways, which were not enriched in our QTL analysis, bear non-silent nucleotide variations between the two strains, resulting in different protein sequences. Therefore, we speculate that most of the quantitative difference in NL storage between AWRI1631 and BY4741 is caused by regulatory or indirect mechanisms that affect the synthesis and storage of NL, rather than by different activities of the metabolic enzymes themselves due to sequence variations.

Our results demonstrate that some cellular processes can have opposite effects in different strain backgrounds. An example for such a behavior is the Rpd3L histone deacetylase complex, with Pho23p as one of its subunits (Fig. 3 and Supplemental Fig. S3), because our data suggest a stimulating role of this complex on lipid storage in AWRI1631 and a repressing function in BY4741. It has to be noted, however, that these results do not allow for a discrimination between a direct effect of the deletion and a possible regulatory mechanism that compensates for the loss of Rpd3L function in BY4741. The substitution of *PHO23* with the allele from the other background indicates that structure and function of the whole complex and its effect on NL storage depends on several factors because the BY4741 strain bearing the AWRI1631 allele of *PHO23* was not altered in its NL content, whereas the NL content of the analogous AWRI1631 mutant was the same as in the strain deleted for *PHO23* (Fig. 4). Another example for the dependence of the effect of an allele on the genetic background is the replacement of *PIG1* from AWRI1631 with the allele from BY4741 (Supplemental Fig. S6) in different segregants with high NL content. The average decrease of the NL content was 6%, but the variation between individual segregants was remarkably high, ranging from + 3.5 to − 15.1%. The high variability of the effect of this substitution in segregants derived from the same parents suggests that the function of Pig1p depends on interactions with several other proteins that are encoded by different alleles in the two parents.

Several additional loci which we did not find during the QTL analysis were retained after seven generations of backcrossing. Since the allele substitutions could not explain the whole extent of variation of NL content between the strains, it has to be assumed that several causal genes were not detected in the QTL study. Interestingly, one single chromosomal region, namely a fragment of the right arm of chromosome II, which showed a broad peak of BY4741-derived genes in the QTL analysis, was also retained in the AWRI line of the backcrossing experiment until the 7th generation. A somewhat different example is a large portion of chromosome I from AWRI1631 that was retained until the 7th generation in the BY line. This region includes a QTL containing *SWH1* and *YAT1* that is much smaller than the retained region, indicating a lack of recombination events on chromosome I in the BY line.

We constructed mutants bearing substitutions of the alleles of *PIG1*, *PHO23* and *RML2* with the alleles of the other reference strain in both backgrounds and in all possible combinations. These efforts were based on the assumption that a switch of the alleles between the strains should cause a change in the NL content of the mutant according to the results in the QTL study. However, our data showed that in most cases such a simple approach is not sufficient to transfer the quantitative contribution of a gene to lipid storage from one strain background to another (Figs. 3 and 4). The most likely explanation for this behavior is that the positive effect of the native proteins on NL accumulation in AWRI1631 depends on additional factors. These factors can be direct interactions with other proteins or indirect effects, for example intracellular conditions like pH or metabolite concentrations. Although the deletion of *PIG1* and *PHO23* indicated a positive genetic interaction for these two genes, this result cannot explain the results of the substitution experiments. Therefore, further studies will be required to elucidate the interaction of these proteins and their function with regard to lipid metabolism.

## Conclusion

As it is the case for all major metabolic pathways, most of the enzymes catalyzing product formation in lipid metabolism are known. However, there is still a lack of understanding with regard to the complete network that determines the activity of this pathway and the storage of a surplus of products as TAG and SE in the lipid droplet. We have shown that QTL analysis can be a powerful tool to uncover novel proteins of this network, especially with regard to strain-specific genetic variations that can have a strong impact on NL storage. Overall, our results confirm that NL storage is a quantitative trait, which is influenced by a large number of proteins, many of them without known connection to lipid metabolism and in some cases in an allele-specific and in a strain-dependent manner.

## Methods

### Media and cultivation conditions

YPD plates (10 g/L yeast extract, 20 g/L peptone; 20 g/L glucose, 20 g/L agar), supplemented with 0.2 mg/mL geneticin/G418 (Calbiochem) or 0.1 mg/mL nourseothricin (Werner BioAgents, Germany) were used for selection after transformations with the respective antibiotic resistance marker.

Yeast cultures were grown in minimal media containing 1.7 g/L Yeast Nitrogen Base w/o amino acids and ammonium sulfate (Difco), 20 g/L glucose as carbon source, 5 g/L ammonium sulfate and 10 mg/L inositol. The medium for lipid analysis and for cell sorting experiments (MM-L/S) was supplemented with the MM^+^ amino acids mixture, as described in [35]. The medium for phenotyping (MM-P) was buffered with 20 mM (2-(N-morpholino)ethanesulfonic acid) (MES) and supplemented with 2 g/L of the following amino acid mixture: 3 g of adenine, 2 g of alanine, asparagine, aspartic acid, cysteine, glutamine, glutamic acid, glycine, isoleucine, methionine, phenylalanine, proline, serine, threonine, tryptophan, tyrosine, uracil and valine, and 10 g of leucine. For recovery of *MATa* segregants, haploid selection (HS) medium was used as described in [36].

Respiratory growth was assessed on YPLac agar plates, consisting of 3 g/L yeast extract, 10 g/L peptone, 0.5 g/L glucose, 0.5 g/L NaCl, 0.6 g/L MgCl_2_ x 6H_2_O, 1 g/L KH_2_PO4, 1 g/L NH_4_Cl, 0.5% KCl·2 H_2_O, 2.2% (vol/vol) lactic acid and 20 g/L agar, pH-adjusted to 5.5 with NaOH.

For lipid analysis, the strains were inoculated from plates into MM-L/S and incubated at 180 rpm/30 °C. 100 μL of the exponentially growing pre-culture were transferred to 100 mL MM-L/S in a shake flask. For the analysis of exponentially growing cells, the cultures were harvested when they reached a density of ca. 2E10 cells/L. Stationary phase cells were harvested 48 h after inoculation.

### X-QTL procedure

A diploid strain derived from crossing Y7092 (isogenic with BY4741, except for the markers required for the synthetic genetic array procedure; therefore, we refer to this strain as BY4741 in the Results section) with AWRI1631 *his3∆::NatMX* was sporulated as described in [37]. Spores were spread on HS medium in 30 Q-tray plates (Fisher Scientific). The colonies were left to grow for 3 days at 30 °C, pooled by scraping them off the plates, incubated in MM-L/S for three hours, and washed with sterile ddH_2_O. 1.5 × 10^8^ cells were incubated with 1 μg/mL Nile Red (Sigma-Aldrich), diluted to ~ 5*10^7^ cells/ml with PBS and sorted in a BD FACS Aria I using PBS as sheath fluid, a 70 μm nozzle and appropriate pressure settings. Cells were gated based on forward/side scatter signals, followed by doublet discrimination to select singlet cells using forward- and side scatter pulse width to area relationship (see Supplemental Fig. S1 for gating strategy). The FL-2 (PE) fluorescence channel (488 nm excitation, 585/42 nm emission, 556 nm long pass filter) served to define the sorting gate (Supplemental Fig. S1; P5), which was set to collect a subpopulation of ~ 2% (~ 3 × 10^6^ cells) with the highest Nile Red fluorescence signals (indicating high NL content). The sorted cells from this gate were spread onto YPD plates (4 × 10^5^ cells per plate). After 48 h growth, the colonies from all plates were pooled. After vigorous mixing, an aliquot corresponding to 10^9^ cells was withdrawn for DNA purification using QIAGEN Genomic-tip 500/G and subsequent DNA microarray analysis and WGS.

#### Identification of potential QTLs with DNA microarrays and the SNPscanner algorithm

GeneChip *S. cerevisiae* tiling microarrays (Affymetrix, USA) were used to detect frequencies of genome regions with non-reference sequence variants in the pools of segregants with average and high NL content, respectively. In addition, DNA isolated from the parental strains was also hybridized, to obtain reference signal values. The DNA microarray experiment was performed as described in [38]. The SNPscanner algorithm [23] was used to identify potential QTLs. Over-representation of one of the strain-specific single nucleotide variations (SNVs) in the subpopulation was detected with the algorithm SNPscanner, which assigns so-called prediction signals to variants between the compared genomes [11, 23], based on which over-representation of a variant in the studied subpopulation of segregants from two parental strains can be established.

To obtain a truly single-nucleotide resolution of the frequencies of SNVs in the subpopulation of segregants with extremely high NL content compared to the average population, WGS of these two subpopulations was performed. Frequencies of SNVs from either parental strain between the two subpopulations was used to identify potential causative genes, as described in [11] (i.e., the X-QTL method).

#### Identification and quantification of QTLs with whole-genome sequencing

Genomic DNA of cell populations with an average NL content and high NL content was sequenced at Donnelly Sequencing Centre (Toronto, ON, Canada) with Illumina HiSeq 2000 using the V3 reagent kit and paired-end 2 × 150 libraries. After demultiplexing the data, FastQC was used to assess the quality of the sequences. Adaptors and bases with quality Q < 20 were trimmed from the reads with the ‘bbduk’ script [39]. Mapping of the reads to the reference *S. cerevisiae* S288C genome (recovered from *Saccharomyces* Genome Database in June 2018) was done with the ‘bwa’ program using the option ‘bwa mem’ and default parameter values [40]. After mapping, the reads were sorted and de-duplicated using Samtools 1.6 [41] and Picard 2.10.2 [42]. Variants were called with the Genome Analysis Toolkit 4.1.4.0 [43] for each strain or population individually and the resulting variants were filtered using the ‘hard filtering’ option. First this was done in haploid mode for both parental strains and the reference genome was corrected with SNVs of each parent, thus producing two mock reference genomes. These were then used as references for SNV calling of population sequences in diploid mode. The number of reads belonging to each allele in SNV positions was reported, the ratio between BY4741 and AWRI1631 alleles was calculated in R for each SNV position and a median of ratios was calculated in 10,000 bp windows in R [44] and visualized using the ggplot2 package [45].

### Strain construction

Yeast strains used in this study are listed in Supplementary Table S1. Standard protocols were used for yeast transformations with the lithium acetate method [46] and for sporulation [37]. Plasmid sequences and genetic modifications were confirmed by Sanger sequencing. Genomic insertions of PCR products by homologous recombination were achieved using primers containing 45–90 bp overhangs for recombination in the target region. For the CRISPR/Cas9-based approaches, two separate transformations were performed. First, the plasmid carrying the Cas9-coding gene was introduced. Second, the resulting strain was transformed with the plasmid encoding gRNA, together with donor DNA [47].

To generate a histidine-auxotrophic AWRI1631 mutant the *HIS3* ORF was replaced with the *NatMX* cassette from plasmid p4339 (kindly provided by Charlie Boone). The loss of histidine prototrophy in nourseothricin resistant colonies was confirmed on -his plates.

#### Gene deletions and gene substitutions

For single deletion mutants the open reading frames of *PHO23*, *PIG1* and *RML2* were replaced with *KanMX4*. For substitution mutants, the ORF and terminator (approx. 400 bp downstream of the stop codon) in the recipient strain were first replaced by a *loxP*-flanked *KanMX4* cassette [48], which was then excised through Cre recombinase treatment. In the donor strain, the *KanMX4* cassette was integrated downstream of the terminator and the region consisting of the ORF and the marker was amplified by PCR and integrated at the same locus in the recipient strain. After successful transformation, the *KanMX* cassette was again removed by Cre recombinase.

Double and triple deletion and some substitution mutants were constructed using CRISPR/Cas9. The *TRP1* selection marker in p414-TEF1^P^-Cas9-CYC1^T^ [47] was replaced with *NatMX6*, resulting in p414-Nat. For the expression of gRNA, the plasmid p426-Kan was obtained by replacing the *URA3* marker in p426-SNR52p-gRNA.CAN1.Y-SUP4t (p426-Ura in this study) with *KanMX*. Plasmids p426-Kan or p426-Ura were amplified by two PCR reactions, each one yielding one half of the vector with identical terminal sequences for homologous recombination and re-circularization by Gibson assembly. One of the primer pairs was designed to introduce the desired recognition sequence at the 5′-end of the gRNA. For deletions, the donor DNA was designed to introduce a premature stop codon. For substitutions, the ORF was first deleted from start to stop codon using DNA from marker-free single deletion mutants as a template, and then the allele from the other parental strain was introduced. Two PAM sequences in the terminator region were selected and each template DNA contained a mutation which removed the PAM sequence.

In the case of *RML2* substitutions, the procedure resulted in strains without functional mitochondria because the intermediate strain, which was deleted for *RML2*, had a *rho*^*0*^ phenotype. Therefore, the strain with the substituted *RML2* locus was crossed with an isogenic wild-type strain of the opposite mating type and haploids with the desired allele and functional mitochondria were selected after sporulation and tetrad dissection.

AWRI1631 *PHO23*^*BY4741*^ *PIG1*^*BY4741*^ was generated by crossing AWRI1631 *PHO23*^*BY4741*^ *with* AWRI1631 *PIG1*^*BY4741*^ after switching the mating type of one of the mutants as described in [20], with a few exceptions. The pHS3 plasmid (Addgene, # 81038) was used, the mating and plasmid loss steps were shortened to an overnight culture in YPD. Ploidy and mating type were tested according to [49].

For backcrossing, the strains AWRI1633 *his3*Δ::*NatMX6*, AWRI1633 *his3*Δ::*KanMX4,* BY4742 *his3*Δ::*NatMX6* and BY4742 *his3*Δ::*KanMX4* were constructed by replacing the *HIS3* ORF with the respective cassettes.

### Phenotyping

Segregants were grown in 96 well plates in 200 μL minimal media (MM-P) at 30 °C without agitation. After 24 h, 20 μL of the cultures were inoculated into 180 μL fresh media and incubated for 72 h. 200 μL of appropriate dilutions of cells in ddH_2_O (OD_600_ below 0.65) were pipetted into black 96 well plates with clear bottom and Nile Red (Sigma-Aldrich, Austria) was added to a final concentration of 5 μg/mL. OD_600_ and fluorescence (excitation 510 nm, emission 585 nm) were measured using a Tecan Sapphire microplate reader (Tecan, Switzerland) before and after 25 min of Nile Red treatment in the dark at room temperature. The NL content was determined as the difference in Nile Red fluorescence before and after the treatment, normalized to the corresponding well’s cell density (OD_600_).

Four replicates of Y7092 and AWRI1631 were included on each plate. The average value for the Y7092 strain was used for normalization, to allow for comparability between plates. Frequency distributions of the fluorescence intensity were analyzed by binning data into 100 equally sized intervals.

### Backcrossing

The F1 generation was obtained from the diploid strain generated by crossing Y7092 with AWRI1631 *his3∆::NatMX*. Due to the genetic markers in these two strains (Supplemental Table S1) only haploid *MATa* strains were able to grow on HS plates [36]. For backcrossing, the *MATα* parental strains AWRI1633 (isogenic with AWRI1631, except for the mating type) and BY4742 (isogenic with BY4741, except for the mating type and *LYS2* and *MET15* loci) were used. A total of 2288 segregants from F1 were analyzed for their FI with the microtiter plate-based assay described above and one of the segregants with high FI and without a flocculation phenotype, strain 11/E6, was selected for the backcrossing with BY4742 *his3∆::KanMX* and AWRI1633 *his3∆::KanMX*. The procedure was repeated for both lineages until generation F7. For F2-F7, ca. 190 segregants per generation were analyzed for their fluorescence intensity. For the segregant selected for the next backcrossing, the resistance marker at the *his3* locus (*KanMX* or *NatMX*) was determined and the strain was crossed with the parental strain bearing the complementary resistance marker at this locus, allowing for the selection of diploids on plates containing both antibiotics. Measurements for segregants with higher FI than AWRI1631 were performed in triplicates. Segregants were genotyped for the presence of the specific alleles using the SNV-specific PCR, as described in [34].

Genomic DNA of strains resulting from backcrossing was sequenced at GATC Biotech AG (Germany) using the Genome Sequencer Illumina HiSeq with 2 × 150 bp Nextera libraries in a multiplexed mode. Quality control of the reads, trimming, mapping and variant calling in haploid mode were performed for the genome of each segregant as described in the section „Identification and quantification of QTLs with whole-genome sequencing “(see above). Mock reference genomes of the parental strains, constructed as described above, were used as references for SNP calling of all backcrossed strains. After removing all SNVs not appearing in either parent (false calls or new mutations), the parental origin of a certain genomic region (BY4742 or AWRI1633) was determined if at least five consecutive SNVs of the region (extending between the furthest two in the line of consecutive SNVs) could be matched to the variants of one parent. Sequencing depth across the genome was calculated with Samtools 1.6 [41]. Segregants with a duplicated part of chromosome XVI were identified based on the higher sequencing depth of the corresponding chromosome part and in these segregants the duplicated part of the chromosome was excluded from the visualization. Data were visualized with karyoploteR [50] and ggbio [51] packages in R [44].

### Lipid analysis

The CDW of the cultures was determined according to [35]. For lipid extraction, a culture volume corresponding to 30–50 mg of CDW was harvested and centrifuged at 3000 rpm for 5 min at room temperature. The pellet was immediately frozen in liquid nitrogen and stored at − 75 °C until processing. Lipids were extracted and analyzed as described in [35].

All data are derived from a minimum of three independent experiments. In the figures presenting results from lipid analyses, the means of the TAG and SE contents and their standard deviations are shown. *p*-values were calculated with an unpaired two-tailed t-test. The replicates were generated on different days, by plating aliquots from the frozen culture stock onto YPD plates, followed by cultivation as described in the section ‘Media and cultivation conditions’. The culture stock of each mutant was derived from a single colony after the last transformation leading to the desired genotype.

## Supplementary Information

**Additional file 1: Supplemental file S1**: Tables S1-S3, Figs. S1-S9

## Data Availability

All WGS data have been deposited in the Sequence Read Archive (https://www.ncbi.nlm.nih.gov/sra) and can be accessed via the bioproject PRJNA644483. All other relevant data are within the manuscript and its Supporting Information file.
