# Supplementary material for: Identification of novel genes involved in neutral lipid storage by quantitative trait loci analysis of Saccharomyces cerevisiae
Source: BMC Genomics. 2021 Feb 9;22:110. doi: 10.1186/s12864-021-07417-4 (PMC7871550; doi:10.1186/s12864-021-07417-4)
Supplement: Supplementary file 1 — Additional file 1: Supplemental file S1: Tables S1-S3, Figs. S1-S9 [file 12864_2021_7417_MOESM1_ESM.docx]

**Table S1:** Strains used in this study

| Strain | Genotype | Source |
| --- | --- | --- |
| Y7092 | *MATalpha can1Δ::STE2pr-Sp_his5 lyp1Δ0 his3Δ1 leu2Δ0 ura3Δ0 met15Δ0* | [1] |
| BY4741 | *MATa his3Δ1 leu2Δ0 met15Δ0 ura3Δ0* | [2] |
| BY4742 | *MATalpha his3Δ1 leu2Δ0 lys2Δ0 ura3Δ0* | [2] |
| AWRI1631 | *MATa hoΔ* | [3] |
| AWRI1631 | *MATa hoΔ his3Δ::NatMX6* | this study |
| AWRI1633 | *MATalpha* | this study |
| BY4741xAWRI1631 | *MATa/MATalpha can1Δ::STE2pr-Sp_his5/CAN1 lyp1Δ/LYP1 his3Δ/his3Δ::NATMX6* | this study |
| BY4741 | *pho23Δ* | this study |
| BY4741 | *pig1Δ* | this study |
| BY4741 | *rml2Δ* | this study |
| BY4741 | *pho23Δ pig1Δ* | this study |
| BY4741 | *pho23Δ rml2Δ* | this study |
| BY4741 | *pig1Δ rml2Δ* | this study |
| BY4741 | *pig1Δ rml2Δ pho23Δ* | this study |
| AWRI1631 | *pho23Δ* | this study |
| AWRI1631 | *pig1Δ* | this study |
| AWRI1631 | *rml2Δ* | this study |
| AWRI1631 | *pho23Δ pig1Δ* | this study |
| AWRI1631 | *pho23Δ rml2Δ* | this study |
| AWRI1631 | *pig1Δ rml2Δ* | this study |
| AWRI1631 | *pig1Δ rml2Δ pho23Δ* | this study |
| BY4741 | *PHO23^AWRI1631^* | this study |
| BY4741 | *PIG1^AWRI1631^* | this study |
| BY4741 | *RML2^AWRI1631^* | this study |
| BY4742 | *RML2^AWRI1631^-KanMX4* | this study |
| BY4741 | *PHO23^AWRI1631^ PIG1^AWRI1631^* | this study |
| BY4741 | *PIG1^AWRI1631^ RML2^AWRI1631^* | this study |
| BY4741 | *RML2^AWRI1631^ PHO23^AWRI1631^* | this study |
| BY4741 | *PIG1^AWRI1631^ RML2^AWRI1631^ PHO23^AWRI1631^* | this study |
| AWRI1631 | *rml2∆::RML2^BY4741^* | this study |
| AWRI1631 | *RML2^BY4741^* | this study |
| AWRI1631 | *PIG1^BY4741^* | this study |
| AWRI1631 | *PHO23^BY4741^* | this study |
| AWRI1631 | *PHO23^BY4741^ PIG1^BY4741^* | this study |
| AWRI1631 | *PIG1^BY4741^ RML2^BY4741^* | this study |
| AWRI1631 | *RML2^BY4741^ PHO23^BY4741^* | this study |
| AWRI1631 | *PIG1^BY4741^ RML2^BY4741^ PHO23^BY4741^* | this study |
| BY4742 | *his3∆::KanMX4* | this study |
| BY4742 | *his3Δ::NatMX6* | this study |
| AWRI1633 | *his3Δ::KanMX4* | this study |
| AWRI1633 | *his3∆::NatMX6* | this study |
| AWRI1631 | *rpd3Δ::KanMX4* | this study |
| AWRI1631 | *sap30Δ::KanMX4* | this study |
| AWRI1631 | *rxt2Δ::KanMX4* | this study |
| AWRI1631 | *sds3Δ::KanMX4* | this study |
| BY4741 | *rpd3Δ* | Euroscarf |
| BY4741 | *sap30Δ* | Euroscarf |
| BY4741 | *rxt2Δ* | Euroscarf |
| BY4741 | *sds3Δ* | Euroscarf |
| BY4741 | *mrpl3Δ* | Euroscarf |
| BY4741 | *mrp7Δ* | Euroscarf |
| BY4741 | *mrpl8Δ* | Euroscarf |
| BY4741 | *mrpl49Δ* | Euroscarf |
| AWRI1631 | *gac1Δ::KanMX4* | this study |
| AWRI1631 | *swh1Δ::KanMX4* | this study |
| BY4741 | *swh1Δ::KanMX4* | this study |
| AWRI1631 | *yat1Δ::KanMX4* | this study |
| BY4741 | *yat1Δ::KanMX4* | this study |

**
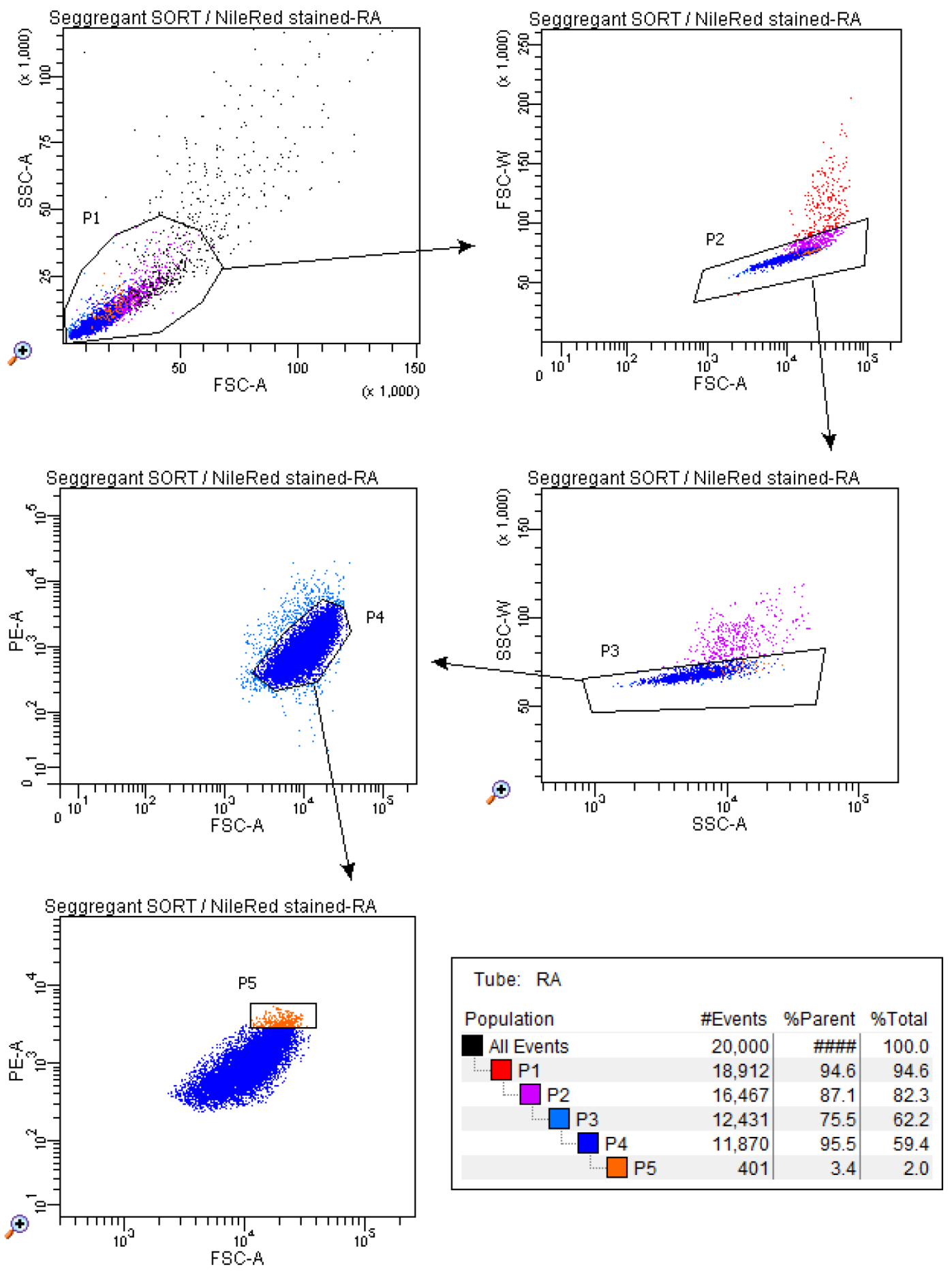
**

**Fig. S1** (previous page): Applied gating strategy to collect cells with high NL content using FACS. Cells were stained with Nile Red (see methods) and applied to FACS analysis. Nile Red fluorescence area intensity (indicating NL content) was detected by using the PE fluorescence channel (PE-A). The depicted gating strategy was used to define a subpopulation of ~2% of total cells with the highest lipid content (gate P5). Cells were gated based on forward/side scatter area signals (FSC-A, SSC-A), followed by doublet discrimination to select singlet cells using forward- and side scatter pulse width (FSC-W/SSC-W) to area relationships.

**Fig. S2** (next page): WGS data showing the median ratios between the frequencies of BY4741 and AWRI1631 parental strain-derived SNVs in the X-QTL analysis along the whole genome. Red points: selected subpopulation with high [NL]. Gray points: non-selected population with average [NL]. The difference between the red and gray datasets is shown with black points. Each point shows a median AWRI1631:BY4741 ratio for all SNVs in a window of 10 000 bp. Higher abundance of the red than of the gray signal (i.e. black signal greater than 0) indicates that this region is enriched for AWRI1631 sequences in the population with high lipid content. Lower abundance of the red signal (i.e. black signal less than 0) indicates the region is enriched for the sequences of the parental strain BY4741 in the population with high lipid content. Regions derived exclusively from the BY4741 parent have no signal due to the absence of SNVs calls in these regions relative to the BY4741 variant calling reference.


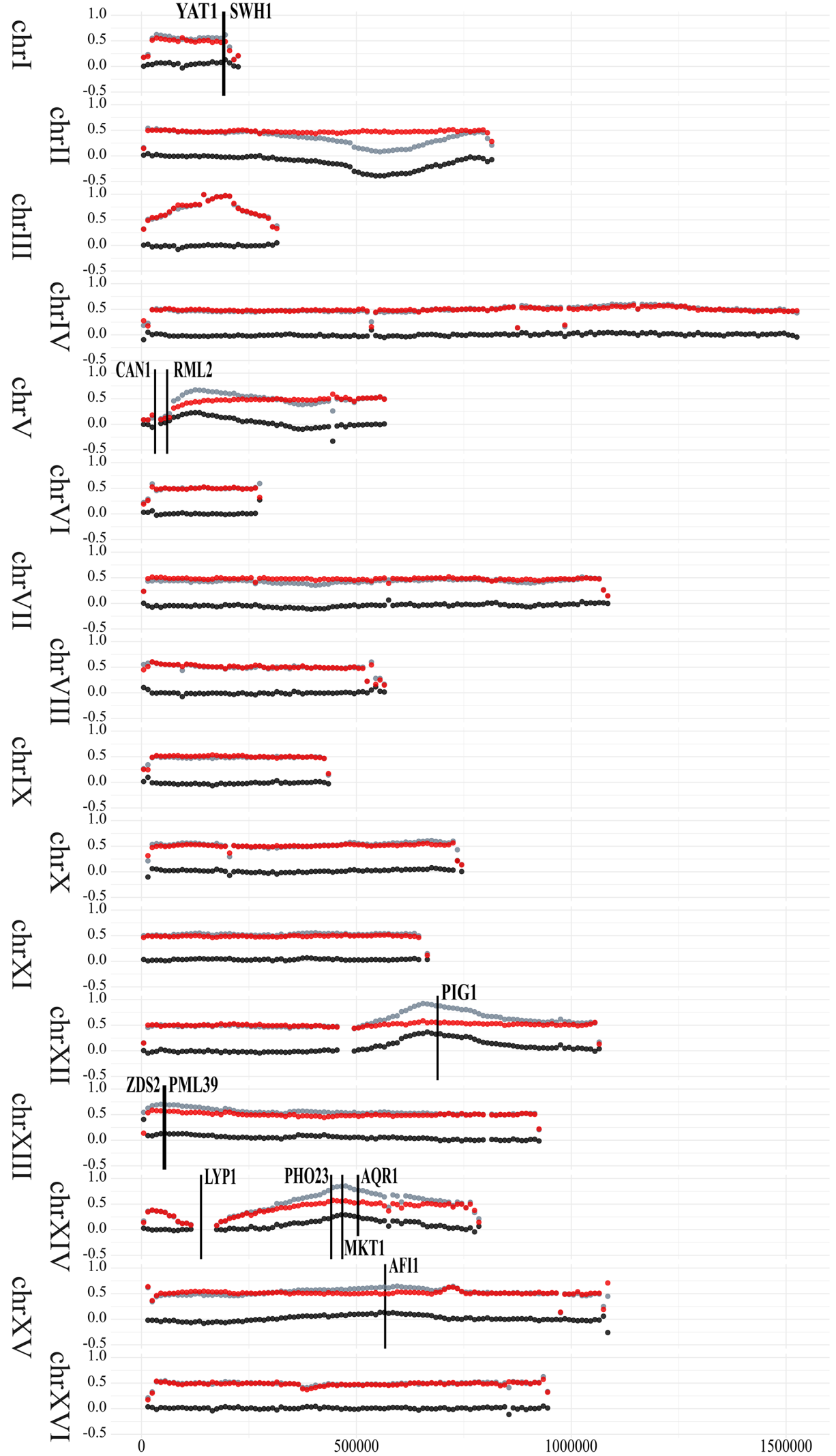





**Fig. S3:** NL content of strains deleted for genes encoding subunits of the Rpd3L complex. In AWRI1631 (panel A) all mutants show the same trend as the strain bearing the deletion of *PHO23*, namely a decrease of TAG content. In BY4741 (panel B), where the deletion of *PHO23* causes an increase in NL content, the deletions of other components of the Rpd3L complex show the same trend, with the exception of *rpd3∆*.

The mutants were cultivated in minimal medium for 48 h. The data are the means from a minimum of three independent experiments and their standard deviations. The p-values are the results of a two-tailed t-test comparing the respective mutant with the wild-type.





**Fig. S4:** NL content of strains deleted for genes encoding subunits of the mitochondrial ribosomal large subunit.

The mutants were cultivated in minimal medium for 48 h. The data are the means from a minimum of three independent experiments and their standard deviations. The p-values are the results of a two-tailed t-test comparing the respective mutant with the wild-type.





**Fig. S5:** NL content of the strains with deletions of *GAC1* and *PIG1*, encoding the two proteins tethering GLc7p to the glycogen synthase Gsy2p. The lipid content of the *gac1*∆ mutant is not significantly different from wild-type (p=0.68, 0.85, 0.46 for total NL, TAG, SE in a two-tailed t-test, respectively).

The mutants were cultivated in minimal medium for 48 h. The data are the means from a minimum of three independent experiments and their standard deviations. The p-values are the results of a two-tailed t-test comparing the respective mutant with the wild-type.


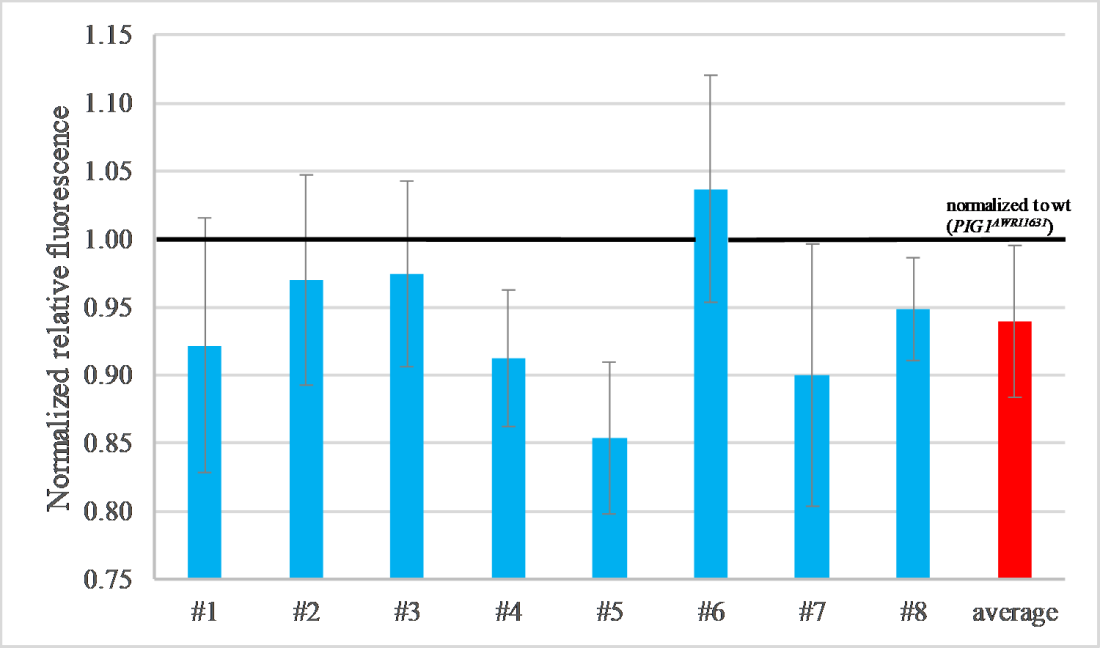


**Fig. S6:** Normalized relative fluorescence of NL-rich segregants after *PIG1* allele swap. From the crossing of AWRI1631 with BY4741, 8 high NL content haploid segregants were selected. Substitution of the superior *PIG1* allele from AWRI1631 with the variant from BY4741 resulted in an average decrease of NL content of 6% (p<0.001 in a two-tailed t-test). Relative fluorescence of each allele-swapped segregant was normalized to the corresponding wild-type segregant. Number of technical repeats was 4, error bars represent standard deviations.

**Fig. S7** (next pages): A) Backcrossing of a segregant from the first generation with the two parent strains, BY4742 (left) and AWRI1633 (right). In each generation, a segregant with high lipid content was selected for sequencing and for the next cycle of mating with the parent of the respective branch, sporulation and germination. Segregant F1 (the same in both branches) followed by F2 to F7 are depicted below the gray bands representing chromosomes. Blue regions indicate parts of the genome originating from BY4741, red regions from AWRI1631. The parental origin of the regions was determined based on SNVs matching one or the other parent. In strains with a duplication of the part of chromosome XVI (as determined by the sequencing depth; data not shown) due to a translocation in the AWRI1631 parent the parental origin of the duplicated part of the chromosome is not shown. In the BY lineage, 6.5% of the AWRI1633 genome were retained, whereas 3.2% of the BY4742 genome were retained in the AWRI lineage. Both values were significantly higher than expected by chance in the 7^th^ generation (p<0.001 and p=0.030, respectively, as determined in a single sample two-tailed t-test). B) Overlap between the X-QTL analysis (black points) and the results of the backcrossing experiment (blue and pink shading). The black points represent the differences between the median AWRI1631:BY4741 allele ratios in 10 000 bp windows of the high-[NL] and average-[NL] populations (see also Fig. S2). A black point signal greater than 0 indicates that the region is enriched for AWRI1631 sequences in the population with high lipid content. A black point signal less than 0 indicates that the region is enriched for BY4741 sequences in the population with high lipid content. Regions derived exclusively from the BY4741 parent have no signal due to the absence of SNVs calls in these regions relative to the BY4741 variant calling reference. Shading denotes the origin from the two parental strains (BY4741 – blue; AWRI1631 – pink) in the F7 backcrossing generation’s segregant with the highest [NL] in the BY lineage (upper ribbon) and in the AWRI lineage (lower ribbon). The parental origin of genomic regions of the segregant was determined in the same manner as for panel A.

A


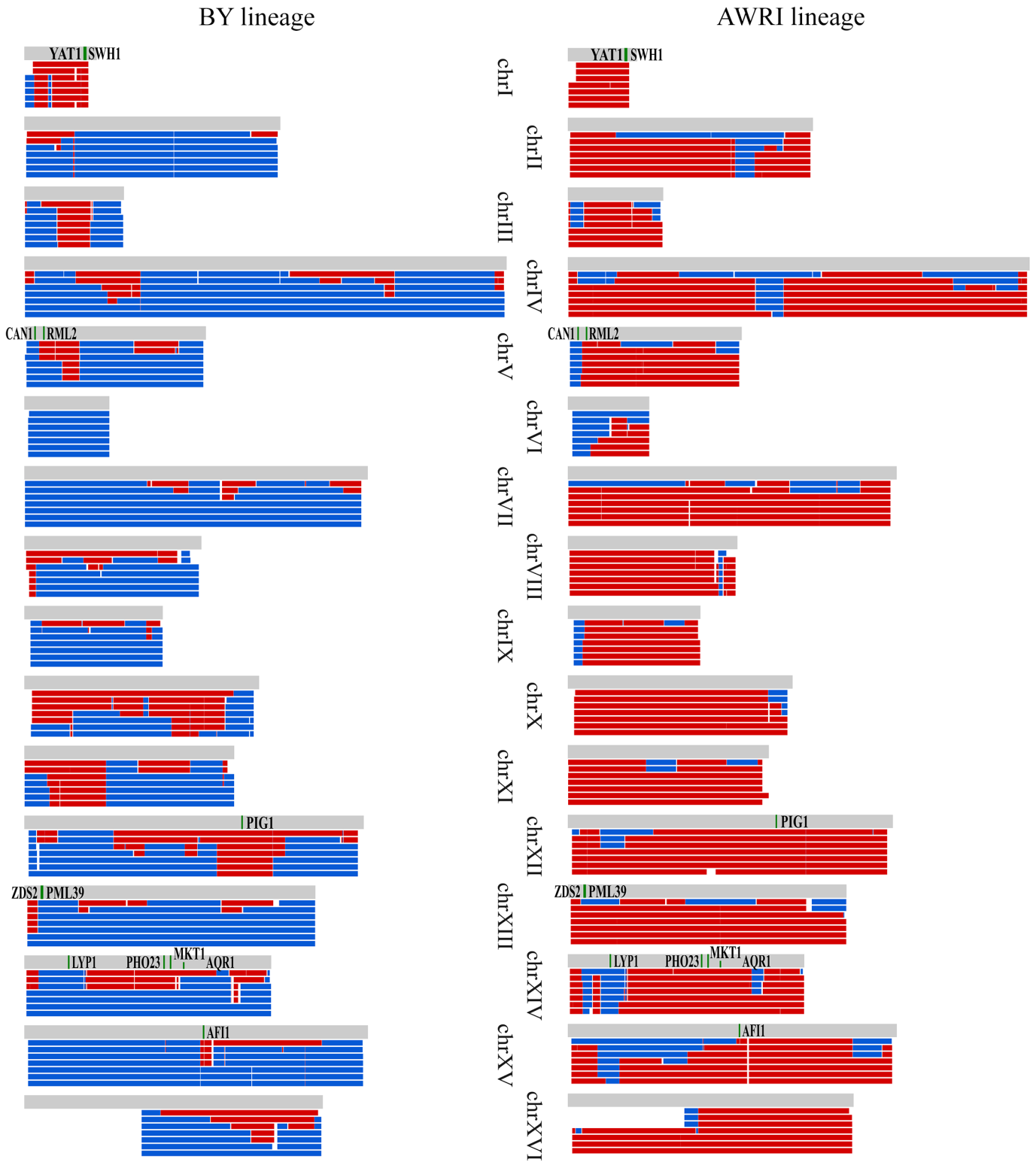


B


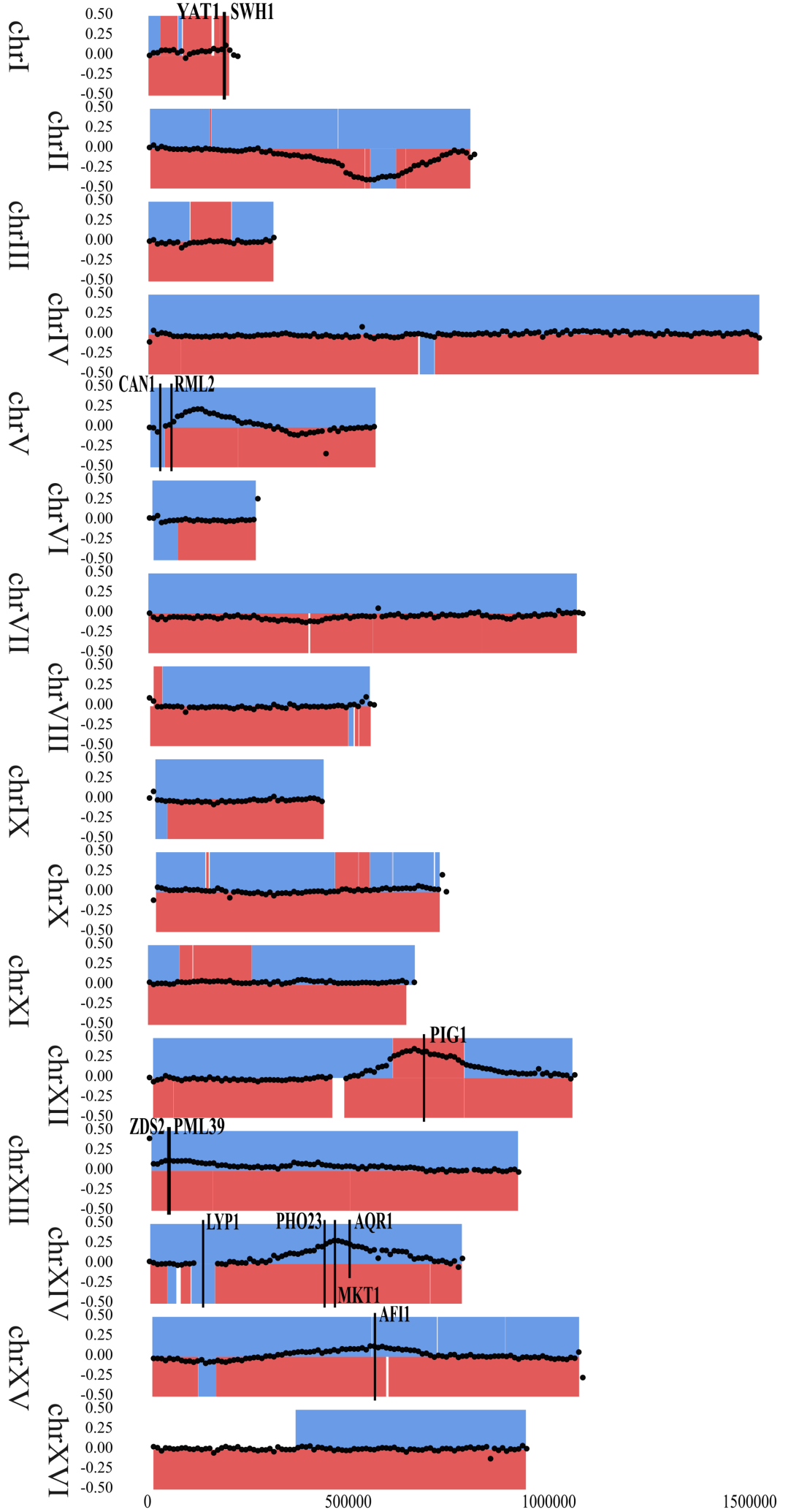





**Fig. S8:** Comparison of the NL content of the wild-type strains (wt) with mutants bearing deletions of *YAT1* or *SWH1*, the two genes at the peak of the QTL region on chromosome I.

A: AWRI1631, B: BY4741. The mutants were cultivated in minimal medium for 48 h. The data are the means from a minimum of three independent experiments and their standard deviations. The p-values are the results of a two-tailed t-test comparing the respective mutant with the wild-type.

**Table S2:** AWRI lineage genes retained in F7 of the backcrossing experiment in the BY lineage

| **Gene Systematic Name** | **Gene Standard Name** | **Amino acid variants (BY4741-AA-AWRI1631)** |
| --- | --- | --- |
|  |  |  |
| **Chromosome I** |  |  |
| *YAL037W* |  | Pro10Ser; Ile11Val; Ile46Ser |
| *YAL040C* | *CLN3* | Ser34Ala; Ala27Val; Arg15Ser |
| *YAL041W* | *CDC24* | Met840Ile |
| *YAL042W* | *ERV46* | Val349Ala |
| *YAL043C* | *PTA1* | Ile568Met; Met526Ile; Ser465Thr; Val176Ala |
| *YAL045C* |  | Asp43Gly |
| *YAL047C* | *SPC72* | Ser549Ala; Val511Leu; Val284Ala; Thr251Ala; Leu238Phe; Thr160Ala |
| *YAL048C* | *GEM1* | Arg316Lys |
| *YAL049C* | *AIM2* | Phe96Leu |
| *YAL051W* | *OAF1* | Leu63Ser; Ile184Thr; Tyr351Asn; Lys594Glu |
| *YAL053W* | *FLC2* | Glu159Asp |
| *YAL054C* | *ACS1* | Ser629Asn; Ala30Ser |
| *YAL055W* | *PEX22* | Gly103Glu; Arg129Lys; Val170Ile |
| *YAL056W* | *GPB2* | Tyr215His; Leu221Ser; Pro269Leu; Ser552Arg; Ala674Thr; Pro777Ser |
| *YAL058W* | *CNE1* | Ala141Val; Asp258Gly; Arg279Gly; Pro300Ser; Pro343Ala; Asn347Lys; Ala349Ser; Pro354Gln; Leu357Pro; Ser409Thr |
| *YAL059W* | *ECM1* | Ala167Glu; Ser171Gly |
| *YAL060W* | *BDH1* | Val172Ile; Ile222Val; Lys284Arg; Ala336Thr |
| *YAL061W* | *BDH2* | Lys19Glu; Glu34Ala; Arg149Gly |
| *YAL056C-A* |  | Asn53Lys; Leu23Phe; Gln13Arg |
| *YAL059C-A* |  | Met7Ile |
| *YAL001C* | *TFC3* | Ser1117Pro; Gly1097Ala; Arg590Gly; Arg258Cys; Arg52Cys |
| *YAL002W* | *VPS8* | Leu49Pro; His374Asp; Val643Ala; Asp672Asn; Asn1011Ser |
| *YAL003W* | *EFB1* | Met182Thr |
| *YAL005C* | *SSA1* | Ala83Gly |
| *YAL007C* | *ERP2* | Ala30Val |
| *YAL008W* | *FUN14* | Gly54Ala; Gln79Glu; Thr85Asn |
| *YAL009W* | *SPO7* | Arg63His; Leu103Phe |
| *YAL010C* | *MDM10* | Ala483Thr; Ser360Leu; Pro343Ser; Ser162Asn; Ala114Gly; Lys83Asn |
| *YAL011W* | *SWC3* | Val326Ile; Ser383Phe; Ser411Ala; Val473Ile; Asp481Gly |
| *YAL013W* | *DEP1* | Leu83Pro; Cys329Arg; Leu365Phe |
| *YAL017W* | *PSK1* | Glu52Lys |
| *YAL019W* | *FUN30* | Ile470Thr |
| *YAL021C* | *CCR4* | Phe408Leu; Glu375Lys; Val12Leu |
| *YAL023C* | *PMT2* | Lys411Thr |
| *YAL024C* | *LTE1* | Ser507Cys |
| *YAL026C* | *DRS2* | Leu76Pro |
| *YAL028W* | *FRT2* | Val525Ile |
| *YAR002W* | *NUP60* | Thr12Ile; His27Arg; Gly158Arg; Thr226Ala; Ser496Pro |
| *YAR007C* | *RFA1* | Arg8Lys |
| *YAL004W* |  | Val141Ile; Ile195Val |
| *YAL019W-A* |  | Pro135Ser |
| *YAL029C* | *MYO4* | Lys1366Asn; Val1293Gly; Ala1209Gly; Ala1071Thr; Val1002Gly; Val926Ala; Ile904Val; Arg864Ser; Ile856Val; Ala736Glu |
| *YAR008W* | *SEN34* | Asp67His; Asp168Asn |
| *YAR014C* | *BUD14* | Ser564Gly; Gly29Cys |
| *YAR018C* | *KIN3* | Arg22Gly |
| *YAR019C* | *CDC15* | Val902Asp; Ser851Thr |
| *YAR023C* |  | Ser142Pro; Asn17Asp; Leu9Val |
| *YAR019W-A* |  | Gly95Glu |
| *YAR023C* | *YAR023C* | Ser142Pro; Asn17Asp; Leu9Val |
| *YAR027W* | *UIP3* | Trp38Arg; Phe105Ser; Lys156Glu; Lys163Glu; His217Asp |
| *YAR028W* |  | Gly73Glu; Val79Met; Met80Ile; Ile83Leu; Met90Leu; Val112Ala; Met137Thr; Lys138Glu; Pro183Ser; Leu193Pro; Tyr197Asp |
| *YAR029W* |  | Glu62Gln; Glu62Ala |
| *YAR031W* | *PRM9* | Tyr8His; Pro43Gln; Leu47Phe; Ser51Asn; Arg52Thr; Phe76Ser; Pro149Ala; Lys193Asn; Gln196Arg; Leu198Ile |
| *YAR033W* | *MST28* | Phe81Leu; Ser94Gly |
| *YAR035W* | *YAT1* | Thr660Ala |
| *YAR042W* | *SWH1* | Thr495Ala; Pro496Ser; Leu558Ile; Val670Ile; Asp1020Gly; Ser1085Leu; Ile1098Val |
| *YAR030C* |  | Val79Leu; Cys47Tyr; Arg16Gln; Gln12Arg |
| *YAR050W* | *FLO1* | Thr19Ile; Ser20Asn; Thr153Ile; Asn174Asp; Asp191Asn; Ile204Val; Tyr214Phe |
|  |  |  |
| **Chromosome II** |  |  |
| *YBL034C* | *STU1* | Leu1217Pro; Gln1090Leu; Ile955Phe; Met933Ile; Asn884Thr; Asp710Ala |
|  |  |  |
| **Chromosome III** |  |  |
| *YCR003W* | *MRPL32* | Ile34Val |
| *YCR006C* |  | Lys147Asn; Ala46Gly |
| *YCR009C* | *RVS161* | Asn88Ser |
| *YCR014C* | *POL4* | Val93Ala |
| *YCR017C* | *CWH43* | Ala905Val; Arg873Lys; Ile634Val |
| *YCR018C* | *SRD1* | Asn96His; Glu24Gly; Met23Ile |
| *YCR021C* | *HSP30* | Val50Ile |
| *YCR023C* |  | Tyr473Cys; Asp229Asn; Leu200Phe |
| *YCR024C* | *SLM5* | Thr6Ala |
| *YCR025C* |  | Thr34Pro; Leu16Pro |
| *YCR026C* | *NPP1* | Ser697Arg; Leu688Pro; Arg439Gly; Glu436Lys |
| *YCR027C* | *RHB1* | Ile92Met |
| *YCR032W* | *BPH1* | Gly203Asp; Ser1289Leu; Asp1785Glu |
| *YCR033W* | *SNT1* | Leu286Phe; Glu482Lys; Ala490Thr; His644Tyr; Ile1023Val; Pro1048Ser |
| *YCR035C* | *RRP43* | Val363Met; Ala102Ser |
| *YCR036W* | *RBK1* | Ala178Thr |
| *YCR038C* | *BUD5* | Leu401Pro |
| *YCR042C* | *TAF2* | Asn1403Lys |
| *YCR044C* | *PER1* | Arg289Gly |
| *YCL007C* | *YCL007C* | Val123Phe |
| *YCR045C* | *RRT12* | Asp357Asn |
|  |  |  |
| **Chromosome VIII** |  |  |
| *YHL033C* | *RPL8A* | Asn252Thr |
| *YHL034C* | *SBP1* | Ala3Thr |
| *YHL035C* | *VMR1* | Met1590Arg; Gly1576Asp; Asp1562Asn; Ala983Val; Pro965Leu; Lys734Arg; Ile700Val; Cys593Tyr; Pro243Leu |
| *YHL036W* | *MUP3* | Leu498Ser |
| *YHL037C* |  | Arg79Cys |
| *YHL038C* | *CBP2* | Gly279Asp |
| *YHL039W* | *EFM1* | Phe43Leu |
| *YHL040C* | *ARN1* | Phe624Ser; Arg353Lys; Asp8Asn |
| *YHL041W* |  | Trp46Ser; Val84Ile |
| *YHL032C* | *GUT1* | Ile193Thr |
|  |  |  |
| **Chromosome X** |  |  |
| *YJL141C* | *YAK1* | Tyr436His; Leu274Pro; Val176Ala; Gln71His; His43Tyr |
| *YJL142C* | *IRC9* | His51Tyr |
| *YJR020W* |  | Asn41Ser |
| *YJR021C* | *REC107* | Pro273Leu; Ser198Cys; Gly147Asp; Asn129Asp; Ser128Asn; Ala43Thr |
| *YJR023C* |  | Leu26Pro |
| *YJR024C* | *MDE1* | Leu119Val; Asn101Asp; Ile63Thr |
| *YJR025C* | *BNA1* | Gly47Asp |
| *YJR030C* | *RBH2* | Lys615Arg; Val609Ile; Thr544Ser; Val541Ile; Asn333Lys; Asp63Gly; Phe11Leu; Glu2Lys |
| *YJR031C* | *GEA1* | Met1033Leu; Leu518Val; Ala279Pro; Ser276Cys |
| *YJR032W* | *CPR7* | Ser87Leu; Val196Met; Leu237Phe |
| *YJR033C* | *RAV1* | Asn1322Lys; Pro1296Ser; Val1205Ile; Ala938Thr; Asn617Asp; Lys501Asn; Glu486Asp; Gln447Leu; Leu332Phe |
| *YJR035W* | *RAD26* | Thr79Ala; Glu211Gln; Gln366His; Gly1003Asp; Asp1076Glu |
| *YJR036C* | *HUL4* | Thr418Ser; Thr410Met; Gln378Lys |
| *YJR037W* |  | Phe77Leu |
| *YJR038C* |  | Leu65Val; Gly45Ser |
| *YJR039W* | *MLO127* | Gly47Arg; Met75Val; Ser237Pro; Cys513Tyr; Thr538Met; Lys555Glu; Gly686Asp; Gly830Asp; Ser864Thr; Phe880Ser; Met924Ile; Phe990Leu; Gly992Ser |
| *YJR040W* | *GEF1* | Gly13Arg; Phe207Leu; Ser257Thr; Ile262Leu; Thr497Ile |
| *YJR041C* | *URB2* | Met1023Ile; Lys840Arg; Met834Ile; Ala778Thr; Met745Ile; Lys704Glu; Ser619Leu; Ser619Pro; Thr607Lys; Arg444Cys; Ala425Thr |
| *YJR042W* | *NUP85* | Asn61Ser; Lys89Arg; Gln243His; Ile433Val |
| *YJR043C* | *POL32* | Ser274Pro; Gly228Glu; Thr223Ala; Thr181Asn; Tyr7Ser; Ala5Thr |
| *YJR045C* | *SSC1* | Gly650Asp |
| *YJR046W* | *TAH11* | Thr145Ser; Ser146Phe |
| *tS(AGA)J* |  | Phe17Ser |
| *YJR019C* | *TES1* | Leu274Ser |
| *YJR049C* | *UTR1* | Val508Glu; Thr492Ile |
| *YJR051W* | *OSM1* | Met348Ile |
| *YJR053W* | *BFA1* | Ile244Val; Arg296Gly |
| *YJR054W* | *KCH1* | Ser458Leu |
| *YJR055W* | *HIT1* | Asn71Lys; Thr112Ala |
| *YJR057W* | *CDC8* | Gly188Asp |
| *YJR059W* | *PTK2* | Asp11His; Ser98Pro; Val106Ala; Asn320Lys |
| *YJR060W* | *CBF1* | Ser84Arg; Cys262Arg |
| *YJR061W* | *MNN14* | Gly542Arg; Lys601Asn; Ile692Leu; Met699Val |
| *YJR062C* | *NTA1* | Ser424Thr; Glu129Gly; Asp111Glu |
| *YJR151C* | *DAN4* | Ser663Phe; Pro231Ser |
|  |  |  |
| **Chromosome XI** |  |  |
| *YKL182W* | *FAS1* | Ile763Val; Glu1216Asp |
| *YKL183W* | *LOT5* | Ala39Gly; Glu127Gly; Asn305Asp |
| *YKL184W* | *SPE1* | Glu44Gly; Leu51Ser; Asp381Asn |
| *YKL185W* | *ASH1* | Ile41Met; Ala311Val |
| *YKL187C* | *FAT3* | Asn724Asp; Ile668Asn; Leu313Val; Asp286Asn |
| *YKL188C* | *PXA2* | Thr814Ile; Arg462Lys; Glu455Asp; Gly104Val |
| *YKL189W* | *HYM1* | Val373Ala |
| *YKL190W* | *CNB1* | Arg33Gly |
| *YKL191W* | *DPH2* | Asp37Asn; Asn242Asp; Met529Ile |
| *tL(UAA)K* |  | Lys8Asn |
| *YKL183C-A* |  | Cys63Trp |
| *YKL192C* | *ACP1* | Pro122Ala |
| *YKL179C* | *COY1* | Arg558Gly; Glu557Gly; Lys520Asn; Ile489Leu; Thr423Ile; Val318Ile; Ser268Asn |
| *YKL097C* |  | Cys104Ser; Leu14Pro |
| *YKL098W* | *MTC2* | Phe160Leu |
| *YKL100C* | *YPF1* | Ser571Ile; Ser489Ile; Thr378Ile; Cys368Tyr |
| *YKL101W* | *HSL1* | Val46Ile; Gly392Glu; Ser464Ala; Lys701Arg; Ala944Thr; Pro1136Arg; Lys1323Arg; Val1400Ile; Asn1439Asp |
| *YKL102C* |  | Leu21Val; Val20Phe; Phe18Ser |
| *YKL103C* | *APE1* | Pro369Ala; Asp328Glu; Asn323Asp; Thr233Ser |
| *YKL105C* | *SEG2* | Ala1107Thr; Asn1004Ser; Glu995Asp; Asn907Asp; Pro868Ser; Thr772Ala; Phe719Leu; Val414Leu; Pro409Ser; Pro404Ser; Gly389Glu; Ser380Arg; Gly298Ser; Gly208Glu; Gly208Arg; Leu66Ser |
| *YKL106W* | *AAT1* | Val44Gly |
| *YKL107W* |  | Arg23His; Lys65Asn |
| *YKL108W* | *SLD2* | Thr166Ala; Lys185Asn; Pro200Ser; Gly298Asp; Lys321Thr; Asp341Asn |
| *YKL109W* | *HAP4* | Ile114Thr; Ser404Pro |
| *YKL110C* | *KTI12* | Ser150Asn; Val4Leu |
| *YKL111C* |  | Phe107Ser |
| *YKL112W* | *ABF1* | Val128Ala; Thr148Ile; Asn279Thr; Thr280Asn; Thr690Asn |
| *YKL114C* | *APN1* | Ser299Asn |
| *YKL115C* |  | Thr67Ile; Tyr18Cys; Leu2Arg |
| *YKL116C* | *PRR1* | Tyr477Asp; Ile294Lys; Cys290Arg |
| *YKL117W* | *SBA1* | Val84Ile; Gln166Lys |
| *YKL119C* | *VPH2* | Val164Ile; Asp119Glu; Lys90Asn; Leu81Ser; Gln59Pro |
| *YKL121W* | *DGR2* | Lys465Arg; Thr504Ala; Ser558Gly; Asn713Asp |
| *YKL122C* | *SRP21* | Ser42Phe |
| *YKL123W* |  | His44Arg |
| *YKL124W* | *SSH4* | Gly533Ser |
| *YKL125W* | *RRN3* | His131Gln; Lys167Glu |
| *YKL126W* | *YPK1* | Val467Ile |
| *YKL127W* | *PGM1* | Lys218Asn; Arg556Lys |
| *YKL129C* | *MYO3* | Ser1203Leu; Met1084Ile; Ile947Val; Ser890Pro; Asp673Asn |
| *YKL132C* | *RMA1* | Arg231Cys; His221Tyr; Arg216Lys; Ser149Asn; Thr133Ala; Ser101Arg; Glu98Lys; Gly53Glu |
| *YKL134C* | *OCT1* | Pro18Leu |
| *YKL136W* |  | Gly3Asp; Phe8Ser; Lys85Glu |
| *YKL137W* | *CMC1* | Gly87Asp; Thr110Ala |
| *YKL138C* | *MRPL31* | Gln52Arg |
| *YKL140W* | *TGL1* | Gly423Asp; Asp473Asn; Gln511Glu |
| *YKL141W* | *SDH3* | Ser13Pro; Leu95Phe |
| *YKL146W* | *AVT3* | Lys511Asn |
| *YKL147C* |  | Asn175Tyr; Gly26Asp |
| *YKL150W* | *MCR1* | Ser240Gly |
| *YKL153W* |  | Gly141Trp |
| *YKL161C* | *KDX1* | Thr308Ile; Pro285Thr; Ser45Leu |
| *YKL162C* |  | Gln316Leu; Ile288Val; Ser259Asn; Lys228Glu; Glu74Asp; Ser28Thr; His13Arg |
| *YKL164C* | *PIR1* | Leu339Val; Ala14Thr |
| *YKL165C* | *MCD4* | Ile387Thr |
| *YKL166C* | *TPK3* | Ile208Thr; His122Arg |
| *YKL167C* | *MRP49* | Gly131Arg |
| *YKL168C* | *KKQ8* | Arg534Gln; Gly352Glu; Ser347Asn; Pro341Ser; Gln79Arg; Asp71Gly |
| *YKL169C* |  | Pro90Ser |
| *YKL171W* | *NNK1* | Thr391Ile; Thr575Arg |
| *YKL173W* | *SNU114* | Thr88Ile; Glu447Lys; Asn451Asp; Ile458Ser; Arg725Lys; Asp742Gly; Ala802Thr |
| *YKL175W* | *ZRT3* | Ala129Thr; Gly149Asp; Arg186Ser |
| *YKL176C* | *LST4* | Ser792Thr; Asn784Ser; Ser700Thr; Val27Ile |
| *YKL162C-A* |  | Gln28Glu |
| *YKL106C-A* |  | Ile6Lys |
| *YKL178C* | *STE3* | Glu314Lys |
| *YKL096W-A* | *CWP2* | Phe11Leu |
|  |  |  |
| **Chromosome XII** |  |  |
| *YLR236C* |  | Leu49Phe; Cys42Arg |
| *YLR237W* | *THI7* | Val7Phe; Ala38Val; Ser66Ala; Val140Leu; Val494Ile; Leu500Ile; Leu501Phe |
| *YLR240W* | *VPS34* | Ser449Thr |
| *YLR246W* | *ERF2* | Leu53Ile; Leu82Phe; Val85Ile |
| *YLR247C* | *IRC20* | Arg1305Gln; Asn1287Asp; Lys1222Arg; Ile1014Thr; Gly829Ser; Glu813Gly; Glu448Asp; Thr430Ala; Thr426Arg; Ile325Arg; Lys173Ile; Asn149Asp; Arg69Gln; Lys18Arg; Ala3Glu |
| *YLR248W* | *RCK2* | Gln113His; Ala456Ser |
| *YLR249W* | *YEF3* | Ile153Phe |
| *YLR251W* | *SYM1* | Leu6Phe |
| *YLR252W* |  | Tyr59Cys; Tyr78Asn |
| *YLR253W* | *MCP2* | Ile430Val |
| *YLR255C* |  | Gly94Trp |
| *YLR256W* | *HAP1* | Thr382Arg; Ser455Asn; Lys1474Glu |
| *YLR257W* |  | Glu105Lys; Lys294Arg |
| *YLR260W* | *LCB5* | Asp114Gly |
| *YLR261C* | *VPS63* | Thr108Ile; Ser107Asn; Thr5Met |
| *YLR263W* | *RED1* | Gln41Glu; Lys324Arg; Ile375Thr; Gly376Arg; Pro406Ser; Ala444Thr; Lys546Thr; Ala635Thr; Met652Val; Cys660Arg; Lys672Asn; Asp673Asn; Gln722His; Ser782Ala |
| *YLR265C* | *NEJ1* | Arg281Cys; Ala270Val; Lys249Glu; Leu231Phe; Glu161Gln; Asn21Ile; Val17Ile |
| *YLR266C* | *PDR8* | Ile601Val; Ala550Gly; Leu371Phe; Thr267Ser; His263Arg; Gly198Asp; Ser17Leu; Lys9Thr |
| *YLR267W* | *BOP2* | Ala107Val; Ile145Thr; Phe194Leu; Asn429Asp |
| *YLR271W* | *CMG1* | Glu32Lys; Arg35Ser; Arg39Cys |
| *YLR272C* | *YCS4* | Leu659Met |
| *YLR273C* | *PIG1* | His636Gln; Thr628Ile; Thr605Asn; Ala579Pro; Lys559Gln; Arg558Lys; Gln510Glu; Ser507Leu; Ser413Pro; Glu372Lys; Gly359Arg; Thr290Ala; Gly184Glu; Tyr178Cys |
| *YLR278C* |  | Thr1305Ile; Ala829Thr; Leu787Pro |
| *YLR280C* |  | Val14Ala; Ala2Val |
| *YLR282C* |  | Cys107Tyr; Leu106Gln; Ile99Val |
| *YLR283W* |  | Met2Ile; His181Arg |
| *YLR284C* | *ECI1* | Met25Ile |
| *YLR285W* | *NNT1* | Asp133Gly; Asp133Glu; Ser138Thr |
| *YLR286C* | *CTS1* | Thr461Lys; Ile434Leu; Pro433Ser; Thr321Ser; Arg23Ser; Leu16Pro |
| *YLR287C* |  | Tyr334Cys; Ser331Asn; Leu241Ser; Asp7Asn |
| *YLR287C-A* | *RPS30A* | Val50Ile |
| *YLR288C* | *MEC3* | Asp454Glu; Gly384Ala; Thr120Ile |
| *YLR289W* | *GUF1* | Ser260Phe |
| *YLR290C* | *COQ11* | Gln28Lys |
| *YLR291C* | *GCD7* | Ser143Gly |
| *YLR292C* | *SEC72* | Arg154Lys |
| *YLR294C* |  | Ser16Phe; Leu3Pro |
| *YLR295C* | *ATP14* | Ser99Thr |
| *YLR296W* |  | Ile69Leu; Ser92Asn; Met102Ile; Thr105Ala |
| *YLR298C* | *YHC1* | Ser195Asn |
| *YLR299W* | *ECM38* | His171Arg |
| *YLR301W* | *HRI1* | Ala63Val |
| *YLR302C* |  | Ser40Phe |
| *YLR305C* | *STT4* | Ser1605Gly; Ser1414Gly; Gly564Ser; Glu284Val; Ser34Asn |
| *YLR307W* | *CDA1* | Ala14Gly; Lys18Glu |
| *YLR308W* | *CDA2* | Gln160His; Thr267Ser |
| *YLR309C* | *IMH1* | Asn833Asp; Ser823Ile; Asp752Glu; Thr478Ile; Thr312Arg; Ala276Thr; Arg261His |
| *YLR310C* | *CDC25* | Gly1579Asp; Gly1579Ser; Thr1157Ala; Ile893Phe; Glu481Lys; Arg286His; Glu279Gly; Glu268Gly |
| *YLR311C* |  | Val76Leu; Val13Leu |
| *YLR312C* | *ATG39* | Gln339His; Thr251Ile; Leu128Arg; Thr127Ser; Val104Ile; Ala80Val |
| *YLR312W-A* | *MRPL15* | Asp169Gly |
| *YLR313C* | *SPH1* | Ala431Thr; Gly400Ser; Ser378Phe; Ala377Thr; Ser316Leu; Val306Glu; Asp281His; Thr141Ser; Ser78Pro; Phe55Leu; Gln41His |
| *YLR314C* | *CDC3* | Ala158Ser |
| *YLR316C* | *TAD3* | Val222Ala; Thr209Ile; Ile117Arg; Val104Ala; Val104Ile; Val96Ala; Val87Ala; Pro81Leu |
| *YLR317W* |  | Pro118Ser |
| *YLR318W* | *EST2* | Val235Ile; Ile237Met; Arg340Leu; Val520Ala; Gln679Arg |
| *YLR319C* | *BUD6* | Met454Ile; Pro441Ser; Thr352Ile; Thr280Ile |
| *YLR320W* | *MMS22* | His1200Tyr |
| *YLR322W* | *VPS65* | Met63Val; Val81Met |
| *YLR324W* | *PEX30* | Gly3Asp; Ser446Gly; Thr505Ser |
| *YLR326W* |  | Gly11Ser |
| *YLR328W* | *NMA1* | Thr143Ile |
| *YLR329W* | *REC102* | Ser41Pro; Gln167His; Leu257Ile |
| *YLR285C-A* |  | His52Tyr |
| *YLR286W-A* |  | Ala12Thr |
| *YLR264C-A* |  | Asn38Tyr; Thr20Ile |
| *YLR235C* |  | Leu51Ser; Gln40Leu; Leu26Phe |
| *YLR330W* | *CHS5* | Asn271Ser; Pro313His; Ser318Gly; Thr334Ala; Val340Ala; Glu404Gly; Thr437Ile; Gly442Arg; Thr651Ser |
|  |  |  |
| **Chromosome XV** |  |  |
| *YOR306C* | *MCH5* | Ile482Asn; Gly466Ala; Ser436Asn; Ser342Gly; Ser159Phe; Ser53Leu; Thr7Lys |

**Table S3:** BY lineage genes retained in F7 of the backcrossing experiment in the AWRI lineage

| **Gene Systematic Name** | **Gene Standard Name** | **Amino acid variants (AWRI1631-AA-BY4741)** |
| --- | --- | --- |
|  |  |  |
| **Chromosome II** |  |  |
| *YBR150C* | *TBS1* | Glu671Asp; Pro399Ser; Ser343Pro; Arg296His; Lys166Arg; Ser66Pro; Glu12Gly; Phe7Ile |
| *YBR158W* | *AMN1* | Gln226Arg; Arg233His; Asp368Val |
| *YBR159W* | *IFA38* | Ser61Thr; Val66Ala; Asp168Asn; Ser232Gly |
| *YBR162C* | *TOS1* | Ala177Asp; Ile7Met |
| *YBR163W* | *EXO5* | Thr5Ala; His10Tyr; Lys277Arg; Lys369Gln; Ala425Thr; Thr515Pro |
| *YBR166C* | *TYR1* | Gln449Lys; Glu443Asp; Asp313Asn; Ser312Gly; Met91Thr; Val56Ala; Ser38Gly |
| *YBR167C* | *POP7* | His58Gln |
| *YBR168W* | *PEX32* | Val192Ile; Ile209Thr; Glu359Gly; Arg389Lys; Asn395Ser |
| *YBR169C* | *SSE2* | Asp614Gly; Asn468Lys; Asn440Ser; Leu192Pro; Ser192Pro |
| *YBR170C* | *NPL4* | Gly364Arg |
| *YBR172C* | *SMY2* | Pro577Ser; Ala576Thr; Gly485Ser; Ile439Thr; Gly289Asp; Glu271Asp; Gly169Ser; Thr53Ala |
| *YBR174C* |  | Val93Glu; Ser74Pro; Glu61Asp |
| *YBR175W* | *SWD3* | Asn287Ser |
| *YBR176W* | *ECM31* | Val36Phe; Ile65Val; His89Tyr |
| *YBR177C* | *EHT1* | Gln384Arg; Glu132Lys; Lys117Glu |
| *YBR178W* |  | Phe51Leu; Ser82Cys |
| *YBR179C* | *FZO1* | Glu662Ala; Gly429Arg; Gln272Glu |
| *YBR180W* | *DTR1* | Arg44Gln; His44Gln; Thr52Ile; Ser168Ala; Gly305Asp; Leu504Phe; Val528Ala |
| *YBR182C* | *SMP1* | Leu189Phe; Cys188Ser; Pro125Ser |
| *YBR184W* |  | Ile119Ser; Leu221Phe; Phe259Leu; Ala298Ser; Ile464Ser |
| *YBR186W* | *PCH2* | Pro147Ser; Val179Met; Ala182Val; Gly197Asp; Cys340Gly; Ile499Val |
| *YBR187W* | *GDT1* | Leu17Phe; Thr22Ala; Leu72Val; Gly152Asp |
| *YBR188C* | *NTC20* | Ile95Val; Asn91Tyr; Ser43Asn; Ser39Leu; Lys31Glu |
| *YBR189W* | *RPS9B* | Ile49Val |
| *YBR190W* |  | Arg2Ser; Val11Ala; Asp91Asn |
| *YBR191W* | *RPL21A* | Ser126Phe |
| *YBR193C* | *MED8* | Pro218Ser; Asn167Tyr |
| *YBR194W* | *AIM4* | Arg3Gln |
| *YBR195C* | *MSI1* | Ala58Thr |
| *YBR197C* |  | Ser171Pro; Val103Ala; Ser68Thr; Lys28Glu |
| *YBR162W-A* | *YSY6* | Lys7Arg |
| *YBR196C-A* |  | Leu15Phe |
| *YBR182C-A* |  | Ala20Val; Thr9Met |
| *YBR199W* | *KTR4* | Phe27Leu; Phe140Leu; Asp200Gly; Asp280Asn; Ser280Asn |
|  |  |  |
| **Chromosome IV** |  |  |
| *YDR114C* |  | Thr17Ala |
| *YDR117C* | *TMA64* | Ser256Gly |
| *YDR118W* | *APC4* | Val430Leu; Glu584Gly |
| *YDR120C* | *TRM1* | Arg517Gly; Ser135Asn |
| *YDR121W* | *DPB4* | Ala150Val |
| *YDR122W* | *KIN1* | Ala566Pro; Glu617Gly; Val807Met |
| *YDR126W* | *SWF1* | Phe51Leu |
| *YDR127W* | *ARO1* | Ala229Thr; Gly1344Glu |
| *YDR128W* | *MTC5* | Val275Ile |
| *YDR111C* | *ALT2* | Val369Ala; Gly331Asp |
| *YDR129C* | *SAC6* | Tyr335Cys |
|  |  |  |
| **Chromosome V** |  |  |
| *YEL060C* | *PRB1* | Ala444Val; Ala227Thr; Val137Met; Arg89Ser; Ser27Asn |
| *YEL061C* | *CIN8* | Val711Ile; Val667Met; Pro288Ser |
| *YEL062W* | *NPR2* | Arg391Ser |
| *YEL063C* | *CAN1* | Val534Ile |
| *YEL064C* | *AVT2* | Lys444Arg; Val396Ile |
| *YEL065W* | *SIT1* | Gly219Asp; Arg538Thr |
| *YEL067C* |  | His194Tyr; Cys171Phe; Pro152Arg; Pro122Ser; Glu31Gly |
| *YEL068C* |  | Leu35Phe; Ile22Thr |
| *YEL069C* | *HXT13* | Ile14Val |
| *YEL071W* | *DLD3* | Tyr349Phe |
| *YEL072W* | *RMD6* | Lys230Arg |
| *YEL073C* |  | Ser107Ala; Ser3Asn |
| *YEL058W* | *PCM1* | Ile189Thr; Val464Met; Glu537Lys |
|  |  |  |
| **Chromosome VI** |  |  |
| *YFL056C* |  | Asn51Ser; Ile47Leu; Thr46Ile; Val46Ile; Gly45Glu |
| *YFL055W* | *AGP3* | Thr21Ile; His43Tyr; Met49Val; Ser131Pro; Gly217Asp; Gly434Val; Lys446Glu; Asp515Asn; Lys539Glu; Leu550Phe |
| *YFL054C* | *AQY3* | Phe303Leu; Arg259Lys; Ala195Gly; Val172Met; Gly125Val; Arg88Pro; His25Asn |
| *YFL053W* | *DAK2* | Arg102Lys; Pro238Ser; Asp264Glu |
| *YFL052W* | *ZNF1* | Arg18Cys; Pro104Leu; Lys144Ile |
| *YFL051C* |  | Asp77Asn; Arg73Ser; Asp65Asn; Lys62Glu; Lys50Gln; Val11Leu |
| *YFL050C* | *ALR2* | Ser841Gly; Asp837Asn; Val655Leu; Ile357Val; Gly323Asp; Ile322Thr; His267Gln; Thr244Ile; Val180Ala; Asn179Ser; Ala161Thr; Asp160Gly; Met155Thr; Ser112Phe; Phe88Ser; Thr87Ile; Gly82Val; Lys63Arg; Pro19Ser |
| *YFL049W* | *SWP82* | Leu19Val; Ser232Asn; Ser596Ala |
| *YFL048C* | *EMP47* | Lys191Thr; Ile125Val; Asn77Lys; Thr52Ala |
| *YFL047W* | *RGD2* | Ser141Gly; Lys423Arg; Ser507Pro; Met583Val; Arg607Gln |
| *YFL046W* | *FMP32* | Asn60Asp |
| *YFL044C* | *OTU1* | Pro115Leu |
| *YFL042C* | *LAM5* | Phe641Leu; Phe617Val; Glu606Lys; Glu602Lys; Glu579Lys; Ile143Thr; Gly42Glu; Thr36Ala |
| *YFL041W* | *FET5* | Ile15Val; Asn230Asp; Asp426Asn; Ala516Val |
| *YFL040W* |  | Tyr85Cys; Leu146Val; Asp225Glu; Ser497Gly |
| *YFL037W* | *TUB2* | Arg446Gln |
| *YFL036W* | *RPO41* | Thr259Ser; Ser485Gly; Thr777Ser |
| *YFL034C-B* | *MOB2* | Pro162Arg |
| *YFL034W* | *MIL1* | Ser109Ile; Cys260Tyr; Val318Ile; Ser436Pro |
| *YFL033C* | *RIM15* | Ile1378Thr; Ser723Thr; Ser609Thr; Asp607Glu; Ile593Thr; Gln503His; His332Leu; Ala7Thr |
| *YFL041W-A* |  | Lys13Arg |
|  |  |  |
| **Chromosome VIII** |  |  |
| *YHR204W* | *MNL1* | Asp205Glu; His512Arg; Asp763Gly |
| *YHR205W* | *SCH9* | Gly39Asp; Thr59Ala |
| *YHR201C* | *PPX1* | Phe284Leu |
| *YHR206W* | *SKN7* | Thr50Asn; Asn315Asp |
|  |  |  |
| **Chromosome IX** |  |  |
| *YIL157C* | *COA1* | Val17Ala |
| *YIL158W* | *AIM20* | Ala4Val; Ile119Thr; Thr170Asn; Arg204His |
| *YIL159W* | *BNR1* | Ala25Val; Ala181Thr; Asp836Gly; Arg870Lys; Val1052Ala; Val1127Ile; Asn1245Lys; Glu1289Asp; Asn1294Ser; Glu1308Lys |
| *YIL161W* |  | Phe13Ser; Arg15Gly; Asp177Val |
| *YIL162W* | *SUC2* | His84Asn; Glu88Gln; Ile132Thr; Pro409Ala |
| *YIL164C* | *NIT1* | Cys182Tyr; Arg176Leu; Lys31Glu |
| *YIL165C* |  | Ile70Leu |
| *YIL169C* | *CSS1* | Pro431Ser; Ser277Ala; Ser256Ala; Val249Gly |
| *YIL156W* |  | Asp179Gly; Ser487Phe |
|  |  |  |
| **Chromosome XII** |  |  |
| *YLR330W* | *CHS5* | Ser271Asn; His313Pro; Gly318Ser; Ala334Thr; Ala340Val; Gly404Glu; Ile437Thr; Arg442Gly; Ser651Thr |
|  |  |  |
| **Chromosome XIV** |  |  |
| *YNL298W* | *CLA4* | Met285Lys |
| *YNL299W* | *TRF5* | Thr153Ala; Ala558Pro |
| *YNL303W* |  | Met62Val |
| *YNL306W* | *MRPS18* | Pro55Ser; Lys157Glu |
| *YNL308C* | *KRI1* | Gln338Glu |
| *YNL311C* | *SKP2* | Thr532Asn; Ser318Phe; Phe213Leu |
| *YNL256W* | *FOL1* | Leu575Pro |
| *YNL257C* | *SIP3* | Phe609Leu; Lys252Asn; Ile209Lys; Gly56Asp |
| *YNL258C* | *DSL1* | His403Leu; Tyr382Asn; Arg341Gln; Ser208Arg |
| *YNL262W* | *POL2* | Phe579Ser; Thr661Ala; Ala1401Thr; Val1671Ile |
| *YNL267W* | *PIK1* | Glu43Lys; Phe53Tyr; Arg362His |
| *YNL268W* | *LYP1* | Met561Val |
| *YNL270C* | *ALP1* | Cys530Trp; Val517Ile; Val367Ile; Met309Val; Ala126Val |
| *YNL271C* | *BNI1* | Ala1862Thr; Met1763Ile; Pro1468Ser; Val1448Ile; Leu1327Met; Ala1316Thr; Leu1264Ser; Asn1263Lys; Asp1224Gly; Asn1208Asp; Pro1201Arg; Glu1197Gly; Lys1045Glu; Val245Met; Ala235Thr; His172Tyr |
| *YNL272C* | *SEC2* | Gly702Arg; Tyr557Phe; Pro430Ser; Asn421Lys |
| *YNL273W* | *TOF1* | Ser766Pro |
| *YNL277W* | *MET2* | Arg301Gly |
| *YNL278W* | *CAF120* | Ile293Val; Thr939Asn; Pro954Leu; Ser1011Pro |
| *YNL279W* | *PRM1* | Thr136Ile |
| *YNL254C* | *RTC4* | Ala224Val |
|  |  |  |
| **Chromosome XV** |  |  |
| *YOL082W* | *ATG19* | Pro203Ser |
| *YOL083W* | *ATG34* | Thr68Ala; Asp99Asn; Thr99Asn; Ile125Met; Asp204Gly; Lys257Glu; Thr276Asn; Asp329Glu |
| *YOL084W* | *PHM7* | Leu22Val; Ile465Val; Cys474Ser; Thr482Ala; Leu530Ile; Met530Ile; Gly785Ser; Val880Leu |
| *YOL085C* |  | Glu14Lys; Gln10Arg |
| *YOL086C* | *ADH1* | Val152Ile; Glu148Gln; Ala59Val; Ile59Val |
| *YOL087C* | *DUF1* | Leu881Val; Arg860His; Ser847Gly; Ala631Val; Ile513Val; Gly62Glu |
| *YOL088C* | *MPD2* | Ser194Asn; Gly189Asp; Glu146Lys |
| *YOL089C* | *HAL9* | Asp983Gly; Ile781Met; Val770Met; Leu747Gln; Val708Met; Ser527Arg; Val426Ile; Gln362Lys; Cys356Tyr; Glu190Gly; Pro104Gln; Val77Ile; His71Leu |
| *YOL090W* | *MSH2* | Lys446Asn; Ala505Thr |
| *YOL091W* | *SPO21* | Asn73Ser; Thr298Ala; Asn380Ser |
| *YOL092W* | *YPQ1* | Ile51Val; Val151Ala |
| *YOL093W* | *TRM10* | Lys7Asn; Val70Ile; Ser139Pro; Arg196Lys |
| *YOL095C* | *HMI1* | Asn663Lys; Ser647Asn; Gly446Ser; Phe264Ser; His201Arg |
| *YOL096C* | *COQ3* | Ala67Thr; Val11Ile |
| *YOL097C* | *WRS1* | Thr311Pro |
| *YOL098C* | *SDD3* | Asp789Glu; Arg18His |
| *YOL099C* |  | Ser141Arg; Ile3Thr |
| *YOL100W* | *PKH2* | Ile68Met; His650Tyr; Gln745His; Lys809Glu; Thr818Ser; Val820Met; Ile922Val |
| *YOL086W-A* | *MHF1* | Asp66Gly |
| *YOL085W-A* |  | Gly64Cys |
| *YOL097W-A* |  | Ser6Cys |
| *YOL102C* | *TPT1* | His176Gln; Ala25Thr |
| *YOL081W* | *IRA2* | His148Asn; Asn149Ser; His302Tyr; Asp306His; Ile567Val; Met671Val; Ile679Asn; Ala753Thr; Pro898Leu; Ser1099Pro; Phe1135Leu; Val1356Ile; Phe1358Ser; Val1507Ile; Met1683Thr; Ser1724Ala; Asn1733Lys; Ser1760Asn; Leu1791Ser; Val2000Phe; Ala2053Thr; Ser2364Pro; Thr2996Asn |

**Fig. S9** (next page): Recombination and reciprocal translocation VIIItXVI in backcrossing segregants. (A) The high level of similarity between conserved motifs at the 3' end of the promoter of *ECM34* on chromosome VIII and of the *SSU1* promoter on chromosome XVI allows for recombination and reciprocal translocation. Our data suggest that a 77 (76) bp long motif is tandemly repeated in *P_ECM34_* on the translocated chromosome to strengthen the expression of the sulphite pump, Ssu1p, in wine yeasts [4]. The locations of primer annealing are shown schematically. (B) Left gel image: The promoter *P_ECM34_* was found in the BY4741 and AWRI1631 parental strains and in the segregants F4-F7 in the AWRI lineage. It was absent in the BY lineage. Right gel: The difference in length of the PCR products is shown. Only one tandem repeat of the promoter motif was present in the strains of the BY series, whereas two repeats were found in AWRI1631 and in the F4-F7 generations of the AWRI lineage. (C) *P_ECM34_*-*ECM34* was found in BY4741 and AWRI1631 parental strains but was absent in all the segregants. PCR product #16 was sequenced as it had approximately twice the expected length. The high level of similarity between both loci allowed annealing of the ECM34_orf_r primer to the 3’-end of the *SSU1* ORF. This in accordance with (D) and the VIIItXVI translocation. Nonetheless, *P_SSU1_*-*SSU1* (and each element separately) was found in all the segregants and parental strains. (D) The translocation *P_ECM34_-SSU1* was found only in the AWRI1631 parental strain and in the segregants of the AWRI lineage, again beginning with F4 (due to the high level of similarity between loci we obtained an alternative, longer PCR product in the other strains). (E) Reciprocal translocation XVItVIII, i.e. *P_SSU1_-ECM34*, was found in the AWRI1631 parental strain and in both backcrossing lineages. Nonetheless, this translocation was present even in segregants that did not show VIIItXVI or *P_ECM34_-SSU1* translocation. Both translocations were absent in the strains of the BY series, suggesting that they were inherited from AWRI1631 in both lineages.


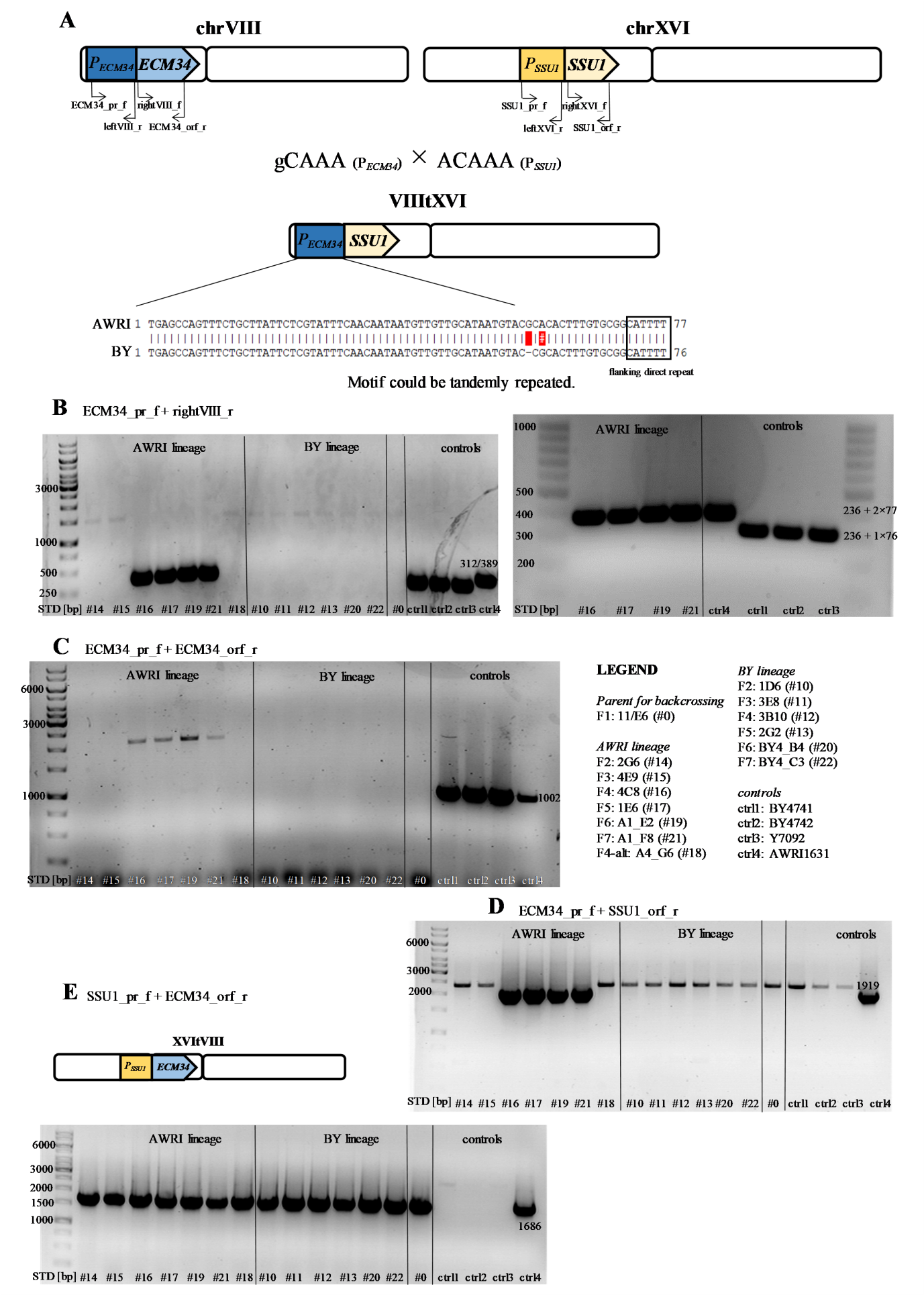


**Literature**

1. Tong AHY, Boone C. Synthetic genetic array analysis in *Saccharomyces cerevisiae*. Methods Mol Biol. 2006;313: 171–92. doi:10.1385/1-59259-958-3:171

2. Winston F, Dollard C, Ricupero-Hovasse SL. Construction of a set of convenient *Saccharomyces cerevisiae* strains that are isogenic to S288C. Yeast. 1995;11: 53–5. doi:10.1002/yea.320110107

3. Borneman AR, Forgan AH, Pretorius IS, Chambers PJ. Comparative genome analysis of a *Saccharomyces cerevisiae* wine strain. FEMS Yeast Res. 2008;8: 1185–1195. doi:10.1111/j.1567-1364.2008.00434.x

4. Pérez-Ortín JE, Querol A, Puig S, Barrio E. Molecular characterization of a chromosomal rearrangement involved in the adaptive evolution of yeast strains. Genome Res. 2002;12: 1533–1539. doi:10.1101/gr.436602
